# Designing Reversible Photoswitching Azobenzene-Modified Nucleotide for Controlling Biological Function

**DOI:** 10.1021/jacs.5c03252

**Published:** 2025-06-13

**Authors:** Juncheng Li, Jinxi Du, Weiwei He, Ibrahim O. Adelakun, Miao Zhong, Savia Boyer, Ya Ying Zheng, Qishan Lin, Serdal Kirmizialtin, Jia Sheng, Ting Wang

**Affiliations:** † The RNA Institute, University at Albany, State University of New York, Albany, New York 12222, United States; ‡ Department of Chemistry, University at Albany, State University of New York, Albany, New York 12222, United States; § Chemistry Program, Science Division, New York University, Abu Dhabi 129188, United Arab Emirates; ∥ Department of Chemistry, New York University, New York, New York 10012, United States

## Abstract

RNA plays vital roles in numerous normal and diseased cellular functions and processes. Reversible photoregulation of oligonucleotide’s structure and function is a powerful strategy for both regulating biological processes and developing novel RNA-based therapeutics. Herein, we designed an azobenzene-modified cytidine phosphoramidite and synthesized a series of oligoribonucleotides containing this photoswitchable residue. We validated the reversible photoisomerization in both ribonucleoside and oligoribonucleotide contexts and studied the overall impact of this cytidine modification through all-atom molecular dynamics (MD) simulations and UV melting experiments. We also showed that the modified oligoribonucleotide can switch the reverse transcription (RT) process upon light irradiation in the presence of various RT enzymes. In addition, the optical control mechanism of the HIV reverse transcriptase-mediated RT process was elucidated by MD simulation. This new chemical biology toolset enables reversible optical control of RNA structures and functions for gene regulation and novel drug development.

## Graphical abstract

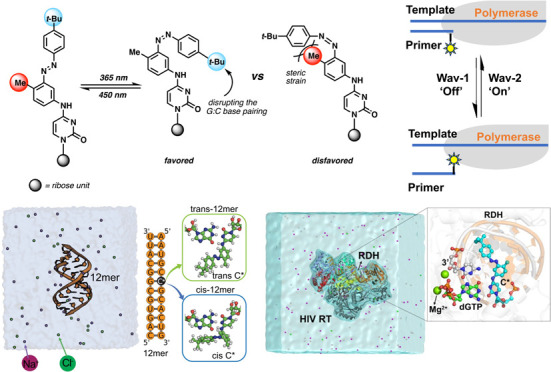

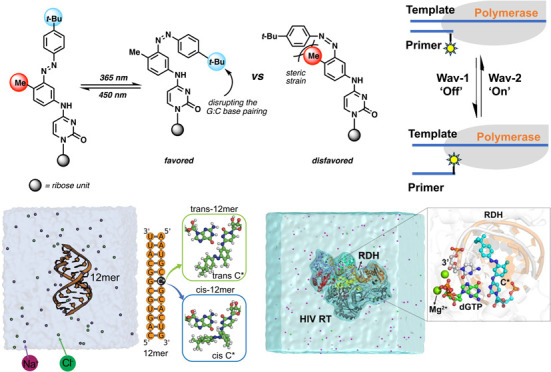

## Introduction

RNA is integral to a wide range of cellular processes, both in normal physiology and in disease states.^1–5^ Controlling RNA activity provides a powerful strategy for regulating processes such as gene expression.^6–9^ In addition, they can be used as a unique strategy to develop novel therapeutics. For that reason, various external stimuli methods, including temperature,^10,11^ pH,^12,13^ small molecule ligands,^14–16^ and metal ions,^17–23^ have been explored to control RNA structures. Among these, light offers significant advantages due to its noninvasive nature and high spatial-temporal resolution, surpassing electrical or chemical stimuli.^24–29^ However, most biomolecules, particularly nucleic acids, lack inherent photochromic properties. Therefore, chemically modified DNA or RNA with suitable photoactive moieties represents a useful way to gain remote control over their structures and function by light.

Earlier light-sensitive biological systems involved the use of photoremovable protecting groups or caging groups.^30^ These groups were used to block key positions on biologically active molecules, allowing activation by exposure to light at a specific wavelength. Despite their utility, this approach is limited by the irreversible nature of the caging group cleavage, restricting their use to one-time activation. In comparison, using photoswitches, a class of compounds that could undergo light-controlled reversible chemical conversions, appears to be a more attractive strategy to control the structures and functions of biomolecules,^28,29^ especially for DNA and RNA owing to the well-developed solid-phase synthesis in the past decades.^31–33^ These compounds are broadly categorized by their mechanisms: azobenzenes, stilbenes, hemithioindigos, and their derivatives belong to the *cis*–*trans* isomerization category, while spiropyrans, diarylethenes (DAE), thiophenefulgides, and their derivatives exhibit reversible open-and-closed states.^28,29^

These photoswitches can be incorporated into nucleoside and sugar–phosphate backbone through different synthetic approaches, such as the attachment of photoswitches on nucleosides, the application of photochromic nucleotides (PCNs), the inclusion of photoswitches in the phosphate backbone, and the replacement of nucleoside by a photoswitch-containing linker.^28,34^ Among these methods, modifying nucleobases, by either attaching a photoswitch or converting to a photochromic nucleoside, is an effective and attractive strategy. This approach minimally disrupts the phosphodiester backbone and is compatible with solid-phase synthesis, enabling efficient and precise placement of photoswitches in oligonucleotides.

As shown in Scheme 1A, Ogasawara and Maeda reported their C8-substituted 2′-deoxyguanosine derivatives for photoregulated hybridization in 2008.^35^ Later, by incorporating stilbenes or azobenzenes, they expanded this concept to guanosine and adenosine.^36^ In 2010, Jäschke lab synthesized diarylethene-functionalized 7-deazaadenosine derivatives, marking the creation of the first photochromic nucleosides (PCNs) based on diarylethene (DAE).^37^ Additionally, both DAEs and azobenzenes photoswitches have been attached to the uracil moiety via an acetylene bridge.^38,39^ More recently, Jäschke lab has expanded DAE-based PCNs to a broader range of pyrimidine nucleosides.^40,41^ In addition, we reported a hydrazone-modified cytidine which could be isomerized with the irradiation of blue light to control DNA replication.^42^

**Scheme 1:**
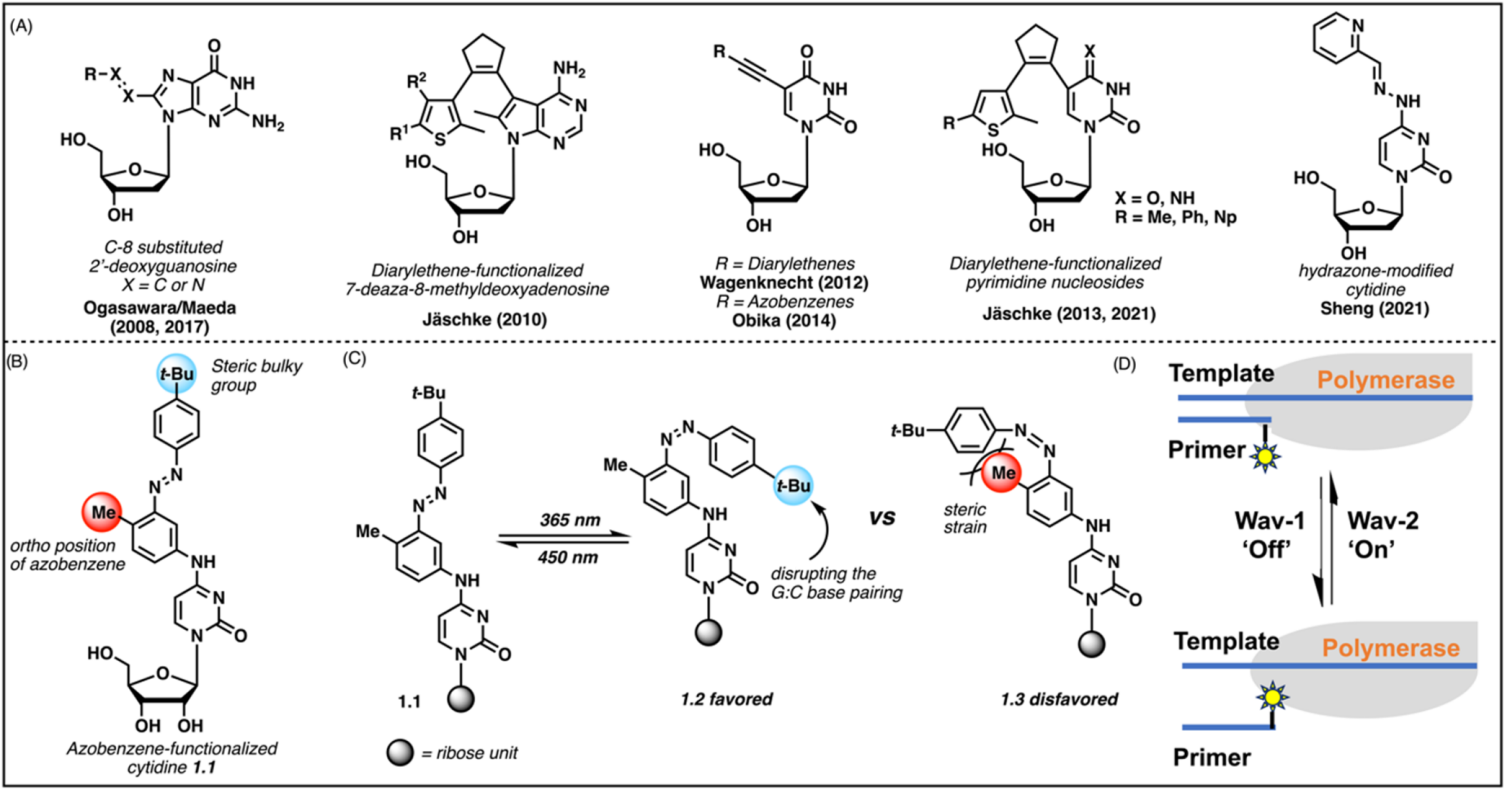
Design of Azobenzene-Modified Cytidine^a^

a (A) State-of-the-art modified nucleobase-derived photoswitches. (B) Our design of azobenzene-modified cytidine. (C) Proposed photoisomerization and the favored conformation for disrupting the G:C base pairing. (D) Biological studies of the photoswitchable oligonucleotide on enzymatic reactions using *in vitro* reverse transcription (RT) with a modified template-directed primer extension reaction.

Continuing our interest in photoactive molecules,^42,43^ we herein report the design and synthesis of azobenzene-modified cytidine, directly attaching azobenzene with adjacent sterically hindered groups at the N4 position of cytosine (Scheme 1B). The sterically bulky *tert*-butyl group is designed to disrupt the hydrogen bonding interactions between the G/C pair upon light irradiation. In addition, the methyl substituent at the *ortho* position of the azobenzene facilitates the desired conformation due to the potential steric strain between the methyl group and the *Z*-azobenzene (Scheme 1C). The modification preserves the capacity of the cytosine base to engage in normal Watson–Crick pairing while employing substituents to alter the pairing stability with the steric effect of the cis isomer. Our design exhibits a pronounced effect on the stability change of dsRNA. Simulation studies confirmed that both substituents are essential for maintaining the photoswitching function by sterically regulating the base pairing. Moreover, we demonstrated the application of this cytidine modification in oligoribonucleotides and illustrated that the elongation activity of reverse transcriptase on RNA templates can be greatly influenced by the structure of a modified nucleoside through light exposure (Scheme 1D). Through all-atom molecular dynamics (MD) simulations, we investigated how this modification impacts the structure, stability, and interactions within the designed nucleic acid motifs upon light exposure. These atomic-level studies provide deeper insights into the photoregulatory activity of our modification and serve as a guideline for the future development of new photoswitchable nucleotides.

## Results and Discussion

The synthesis of the target photoswitchable cytosine analog **2.5** commenced with silylation of the 5′- and 3′-hydroxy groups of cytosine **2.1** with di-*tert*-butylsilyl ditriflate followed by 2′-protection with a TBDMS group (Scheme 2). Appel reaction in the presence of PPh_3_ and CH_2_Cl_2_/CCl_4_ converted compound **2.2** to **2.3** in 77% yield. The coupling of substituted aniline **2.4** (synthesis of compound **2.4** is shown in Figure S1) and compound **2.3** efficiently provided the modified nucleoside **2.5** as a bench stable intermediate (Scheme 2A). UV–vis absorbance experiments have shown that the synthetic molecule **2.5** was able to reversibly switch from the trans conformation to the cis conformation upon different light irradiation (Scheme 2B). NMR studies of **2.5-** ***trans*** and **2.5-** ***cis*** validated the reversible conformational transformation (Supporting Information).

**Scheme 2:**
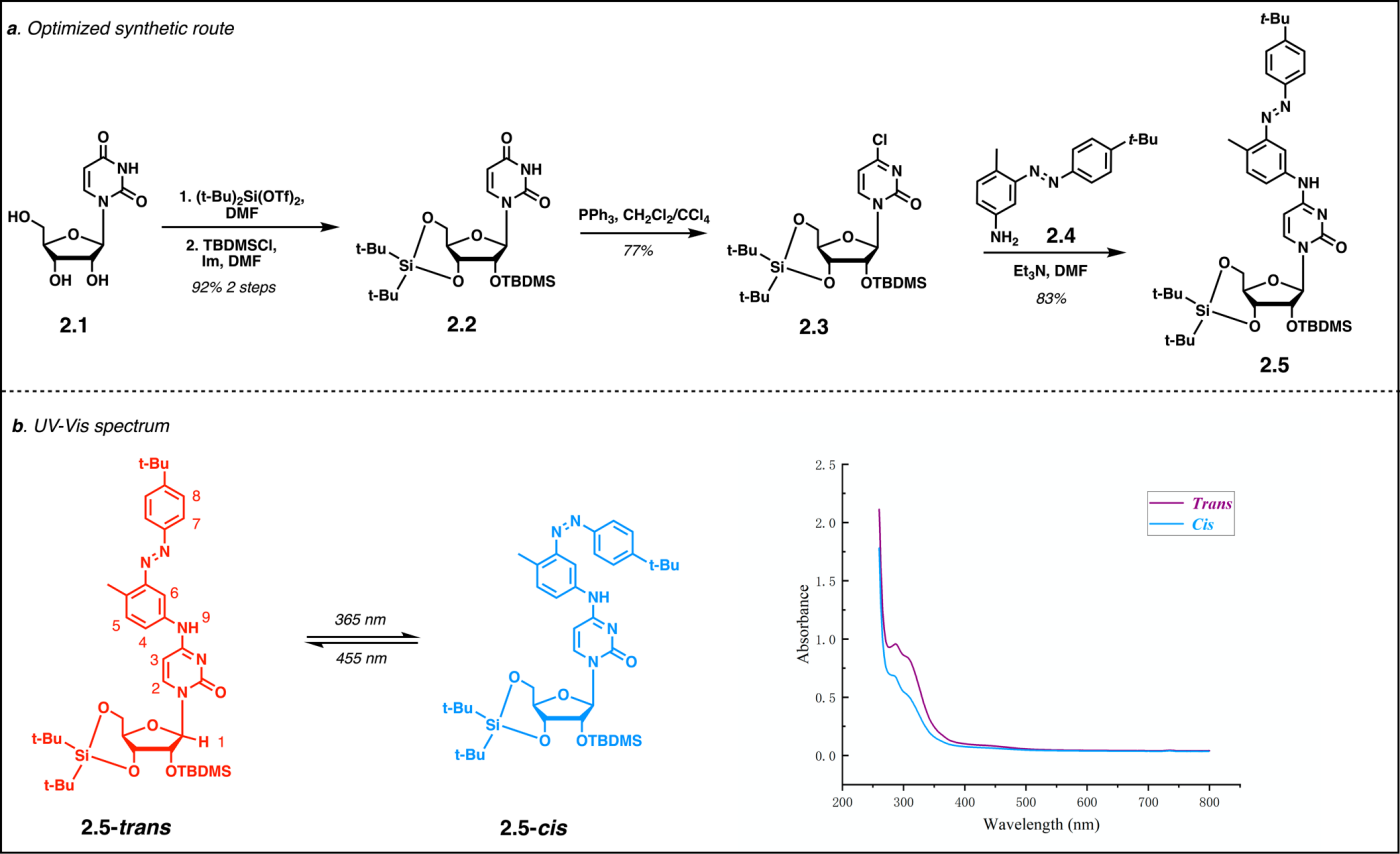
Synthesis of Azobenzene-Modified Cytidine^a^

a (A) Optimized synthetic route. (B) UV–vis spectrum of modified nucleoside **2.5** in both trans and cis conformation.

With the intermediate **2.5** in hand, desilylation using hydrogen fluoride in pyridine, the tritylation with trityl chloride (DMTrCl) at 5′-position, followed by a regular phosphitylation reaction generated the final photoswitchable cytosine phosphoramidite building block **3.1**. The modified cytosine was incorporated in RNA solid-phase synthesis to provide six RNA strands, which were purified by high-performance liquid chromatography (HPLC) and characterized by Orbitrap Mass (Scheme 3).

**Scheme 3:**
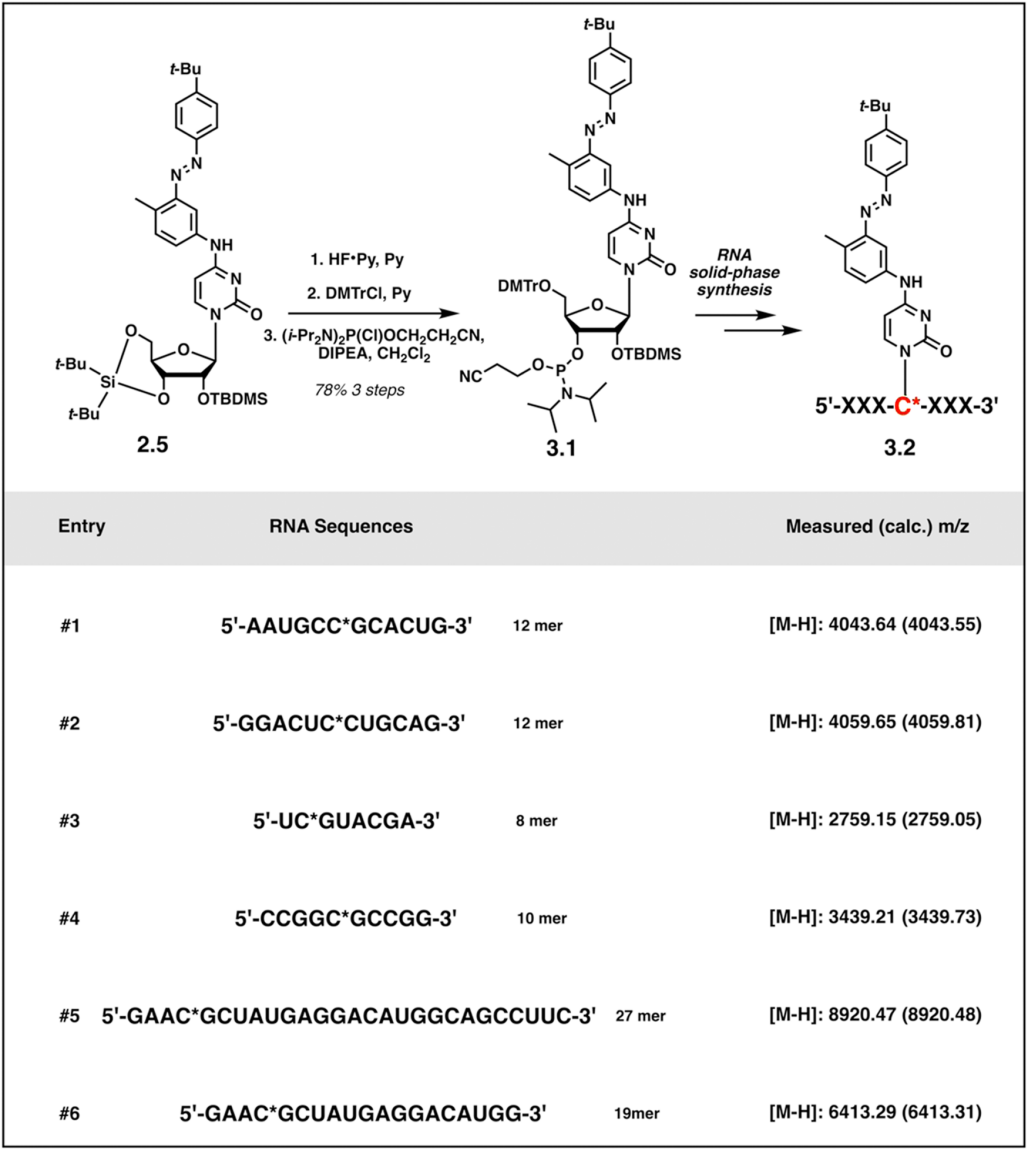
Synthesis of Oligoribonucleotides

Following ethanol precipitation and desalting, the oligo RNA strands were further purified using high-performance liquid chromatography (HPLC). The HPLC profile revealed two distinct peaks: a *cis* isomer peak with a retention time of 10.5 min and a *trans* isomer peak with a retention time of 12.4 min, which is confirmed by back-and-forth illumination. Analysis of these peaks indicates that the freshly synthesized modified RNA sequence 5′-AAUGCC*GCACUG-3′ consists of 87.3% *trans* isomers and 12.7% *cis* isomers (Figure S2). To verify the multiple reversibility of our photoswitch on RNA sequence, we employed two sets of light: a Thorlabs 365 nm mounted light-emitting diode (LED) and a Thorlabs 455 nm mounted LED to irradiate the sample alternately for 5 min each back and forth for 5 times (Figure 1). The conversion ratio in each circle was monitored by the HPLC spectrum. 86% of the sample was converted to *cis* isomer under the first irradiation of 365 nm light, and then 66% of the sample was turned back to *trans* isomer under irradiation of 455 nm light. After two back-and-forth circles, the third irradiation under 365 nm still keeps an 84% conversion yield, suggesting the modification works reliably as a reversible switch in RNA systems.

**Figure 1:**
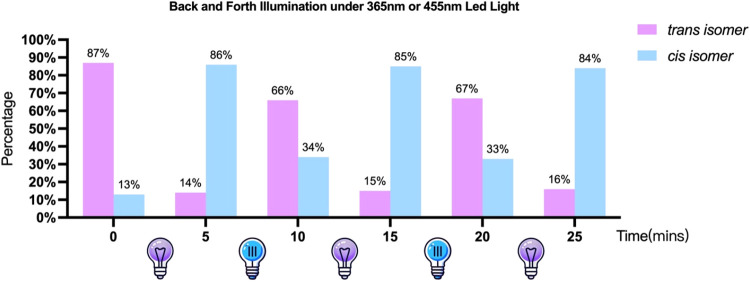
Ratio of *trans* isomer and *cis* isomer during the back-and-forth illumination under 365 or 455 nm LED light alternately with 5 min intervals.

Next, we investigate how the designed modification impacts the RNA structure and dynamics using MD simulations (details provided in the Supporting Information). Independent simulations of the duplex were performed with a modified nucleobase at position 4 locked in either the *trans* or the *cis* conformation (Figure 2A,B). Results were compared with unmodified duplex, denoted as wild type (WT) henceforth. Since the RNA duplex remained folded at room temperature, we focused on the conformational states sampled around the folded state. We investigate the *t-*Bu group conformations, hydrogen bonding patterns of the RNA, and base stacking interactions.

**Figure 2:**
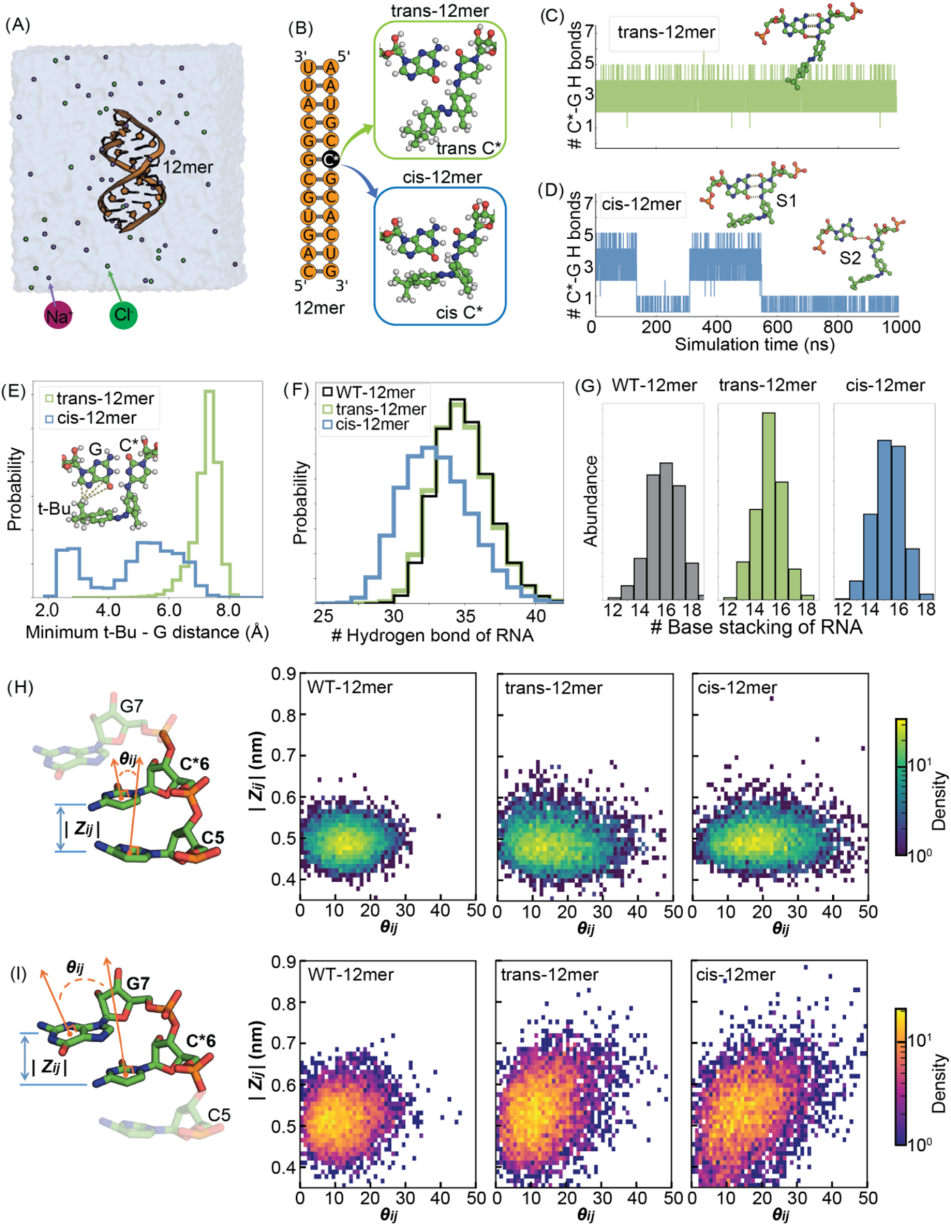
Differences in structural propensities and interactions between 12mer RNA with modifications. (A) Simulation system with 12mer RNA, explicit water, and ions rendered using PyMOL. (B) *trans-* and *cis*-modifications on the 12mer RNA duplex at position C6, denoted as C*. (C, D) Time evolution of the total number of H-bonds of C* with its contacts. For the *cis*-12mer, modified cytidine dynamics is partitioned between fully paired (S1) and partially paired (S2) states. The insets show the two representative configurations. (E) Probability distribution of the minimum distance between the *tert*-butyl (*t*-Bu) and the adjacent guanine base. (F) Probability distribution of the total H-bonds of the RNA duplex in each simulation, with averages of 34.50 ± 0.01 (WT), 34.30 ± 0.03 (trans), and 32.5 ± 0.2 (cis). (G) Population analysis of the total base–base stacking for WT-12mer, *trans*-12mer, and *cis*-12mer. (H, I) Stacking angle and distance distributions between the modified base and its adjacent neighbors for WT-12mer, *trans*-12mer. and *cis*-12mer. (H) Distribution of stacking angle (θ*_ij_*) and base height (*Z_ij_*) between C*6 and C5. (I) Distribution of stacking angle and base height between C*6 and G7.

Comparisons revealed distinct dynamics between the *trans* and *cis* states of the duplex. At the trans state, the *t*-Bu group is locked to a unique state where the modified cytidine maintained stable hydrogen bonding with pairing guanosine, preserving the base-pairing structure (Figure 2C, S1 state). Conversely, in the *cis* isomer, *t*-Bu group exhibited a broader range of conformational states (Figure 2D). These included an S1-like state, where the azobenzene group maintained a fully paired C*–G, similar to the *trans* conformational states, and a disrupted hydrogen bonding state (S2), characterized by *t*-Bu group excursions on the RNA surface. Transient contacts with nearby guanines led to the destabilization of the hydrogen bonding network of the overall RNA duplex (Figure 2E) resulting in changes in the total number of hydrogen bonds between states (Figure 2F). The modification minimally impacted the hydrogen bonding and base stacking interactions at the *trans* state compared with the wild type (Figure 2F,G). In the *cis* state, the number of hydrogen bonds dramatically changed while the base stacking interactions remained minimally affected (Figure 2G). The modification introduces minor perturbations to base stacking, manifested as shifts in the stacking angle (θ*_ij_*) and base height (*Z_ij_*) in the *cis*-12mer case, while the *trans*-12mer shows stacking distributions that more closely resemble those of the WT-12mer (Figure 2H,I). Further simulations of *cis*-12mer derivatives also confirm that both the *ortho*-methyl and the *t*-Bu substituents are essential for maintaining the photoswitching function by locally regulating base pairing; specifically, the *ortho*-methyl group restricts the orientation of the *t*-Bu group, directing it inward toward the base pair and thereby interfering with hydrogen bonding (Figures S7 and S8). While the *ortho*-methyl group is essential for conformational states, we observe a no-major effect of the bulky groups on the electronic properties (Figure S8).

To investigate the effect of *tert*-butyl azobenzene modification on RNA duplex stability and to validate our simulations, we employed UV melting experiments, which directly report on the duplex thermal stability. The melting curve of the RNA oligonucleotide sequence with and without modification is measured. In addition, we repeat the experiment for the modified RNA after 1 h of 365 nm UV illumination (*cis* state). Results are shown in Figure 3 for comparison. Interestingly, the melting temperature showed a reduction for the modified sequences in general with a change of Δ*T* ≈ −7.1 °C for the *trans* state, suggesting a stability difference of Δ*G* ≈ 4.6 kcal/mol. The largest change happened in the *cis* state where we observe a melting temperature reduction of Δ*T* ≈ −24.7 °C, corresponding to a thermal stability difference of Δ*G* ≈ 14.4 kcal/mol relative to the wild type. This result aligns well with our simulation results (Figure 2F) where a reduction in RNA stability was inferred from the changes in the number of hydrogen bonds between *cis* and *trans* states. In conclusion, the azobenzene modification affects the overall duplex stability by changing the hydrogen bonding of RNA. These changes are minor for *trans* configuration, but they significantly impact the stability of RNA at the light-sensitive (*cis*) state.

**Figure 3:**
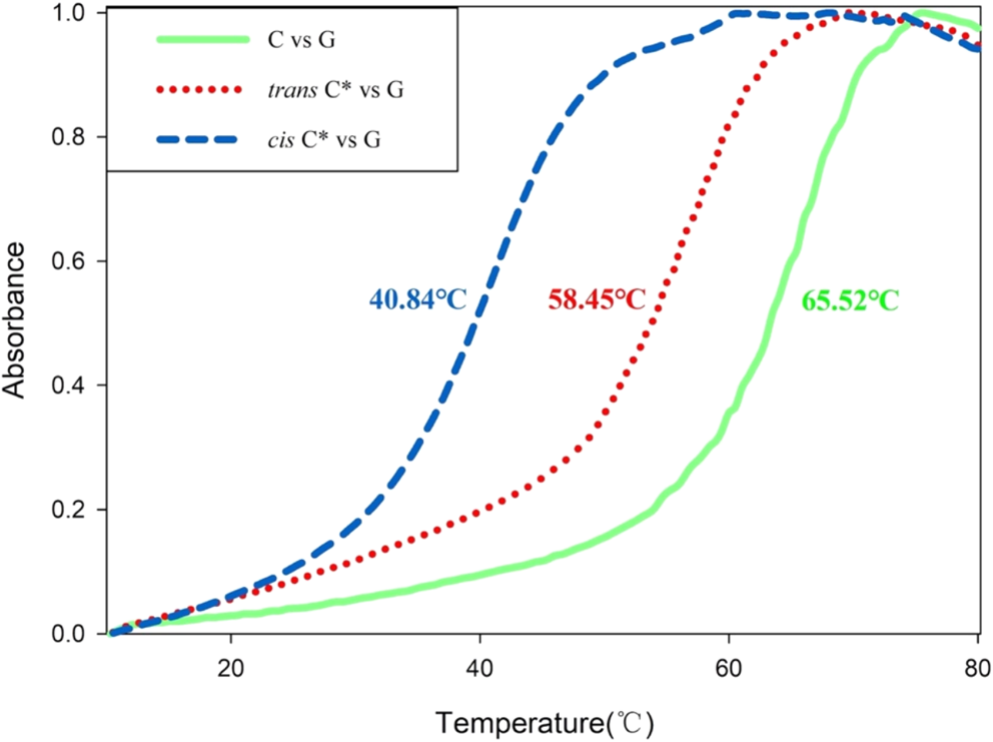
UV-melting curves of the RNA duplex in the wild type and modified sequence. The stability of modified RNA duplexes is assessed by comparing the melting curves of wild type and modified sequence 5′-AAUGCC*GCACUG-3′ (C*) and native 3′-UUACGGCGUGAC-5′ (G). The green solid line represents the *T*_m_ of native C/G (65.52 °C). The red dashed line is for modified C*:G (58.45 °C). The blue dashed line is for modified C*:G, where sequence C was irradiated by 365 nm for 1 h prior to mixing with sequence G (40.84 °C).

To test the possibility of optical control of biological function with the photoswitchable cytidine, we designed a primer extension experiment in which we studied the reverse transcription. A 19nt-modified RNA strand was prepared as a template to complementary base pair with the 5′ end of DNA primer, which was labeled with the fluorescent FAM group. The modified cytidine was placed at the starting site of the reverse transcription experiment as shown in Figure 4A. We first applied Avian Myeloblastosis Virus Reverse Transcriptase (AMV RT) to examine the effect of the modified residue in this biological process. We quantified the yield and fidelity of reverse transcription by fluorescence gel images with a single nucleotide resolution.

**Figure 4:**
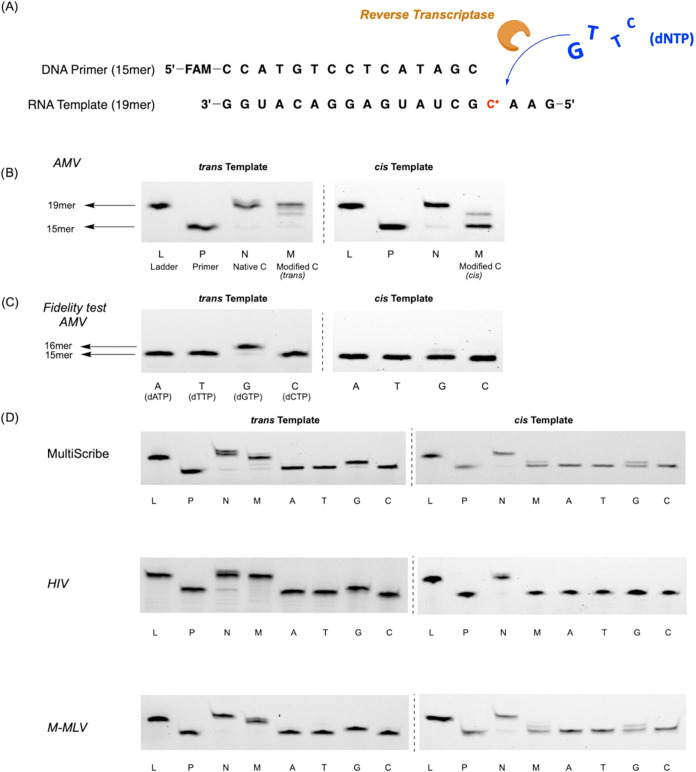
Fluorescent gel image of reverse transcription (RT) reaction. (A) RT reaction with 15mer as primer and 19mer as template and full-length product. (B) RT experiments in the presence of AMV and all of the natural dNTPs. (C) Fidelity test: RT experiments in the presence of AMV and single nucleotide. (D) RT experiments in the presence of other reverse transcriptases including MultiScribe, HIV, and M-MLV. The interpreting error bars of RT experiments are in Figures S3–S6. L: 19mer DNA ladder for reference; P: 15mer DNA primer for reference, N: RT was performed on native 19mer RNA template, M: RT proceeded on modified 19mer RNA template, A, only dATP; T, only dTTP; G, only dGTP; C, only dCTP, AMV: Avian Myeloblastosis Virus Reverse Transcriptase; MultiScribe: recombinant Moloney Murine Leukemia Virus Reverse Transcriptase, HIV: Human Immunodeficiency Virus Reverse Transcriptase, M-MLV: Moloney Murine Leukemia Virus Reverse Transcriptase.

In the controlled group, we conducted a reverse transcription for 1 h in an incubator at 37 °C with a 19nt native RNA template and a 15nt DNA primer in the presence of AMV and natural deoxynucleotide triphosphates (dNTPs). The fluorescent gel images indicated that the 15nt primer was successfully extended to 19nt in high yield. In the treatment group, we performed reverse transcription using a modified RNA template. The modified nucleobase with no light exposure ensures the *trans* state. The fluorescent gel images showed that the primer was still able to complete the extension, which is consistent with the results from the control group. In contrast, when we irradiated RNA under 365 nm UV light for 20 min prior to reverse transcription, the reverse transcription was completely inhibited, resulting in the absence of the full-length products. The observation indicated that the light-activated modification halted the transcription (Figure 4B).

To address the potential concern of whether the halt in transcription is due to the mispairing of the modified template, we designed another controlled group to investigate mispairing by substituting the natural dNTPs with a single nucleotide (dATP, dTTP, dGTP, and dCTP) while keeping the other conditions the same. After reverse transcription, the fluorescence gel images indicated that when the modification was in the *trans* configuration, only the trail containing dGTP extended the primer to 16nt, while the other nucleotides failed to do so. In contrast, when the modification was in the *cis* configuration, extension with any nucleotide was disrupted, and the DNA primer stayed at 15nt length. These results suggested that the introduced azobenzene allows the modification to obey the typical Watson–Crick principle, ensuring faithful base pairing between modified C and native G and clarifying the accuracy of reverse transcription (Figure 4C). To examine the enzymatic compatibility of the modified RNA template, we also screened several common reverse transcriptases, including Human Immunodeficiency Virus Reverse Transcriptase (HIV RT), Moloney Murine Leukemia Virus Reverse Transcriptase (M-MLV RT), and MultiScribe Reverse Transcriptase. Coherent images similar to those of AMV RT (Figure 4D) indicate that the modified RNA template successfully facilitated the reverse transcription under controlled conditions; however, exposure to UV light completely inhibited this process. While maintaining high accuracy, our approach remained compatible with various reverse transcriptase systems.

We employed atomistic simulations to elucidate the mechanism by which azobenzene modification enables the optical control of reverse transcriptase activity. HIV RT enzyme was selected to study the mechanism of the light-controlled transcription process. For that purpose, the enzyme complexed with the RNA:DNA hybrid (RDH) and an incoming nucleotide (dGTP-Mg^2+^) mimicking our experimental setup was simulated in the wild type, in the *trans* and *cis* states independently (Figure 5A,B) (details of simulation setup are in the Supporting Information). The last frame of the simulation data was used to illustrate the interactions of cytidines with the enzyme residues at equilibrium. The interaction between the cytidine nucleotide and the residues in the HIV RT enzyme is shown using two-dimensional (2D) ligand–protein interaction diagrams generated by LigPlot^44^ (Figure 5C).

**Figure 5:**
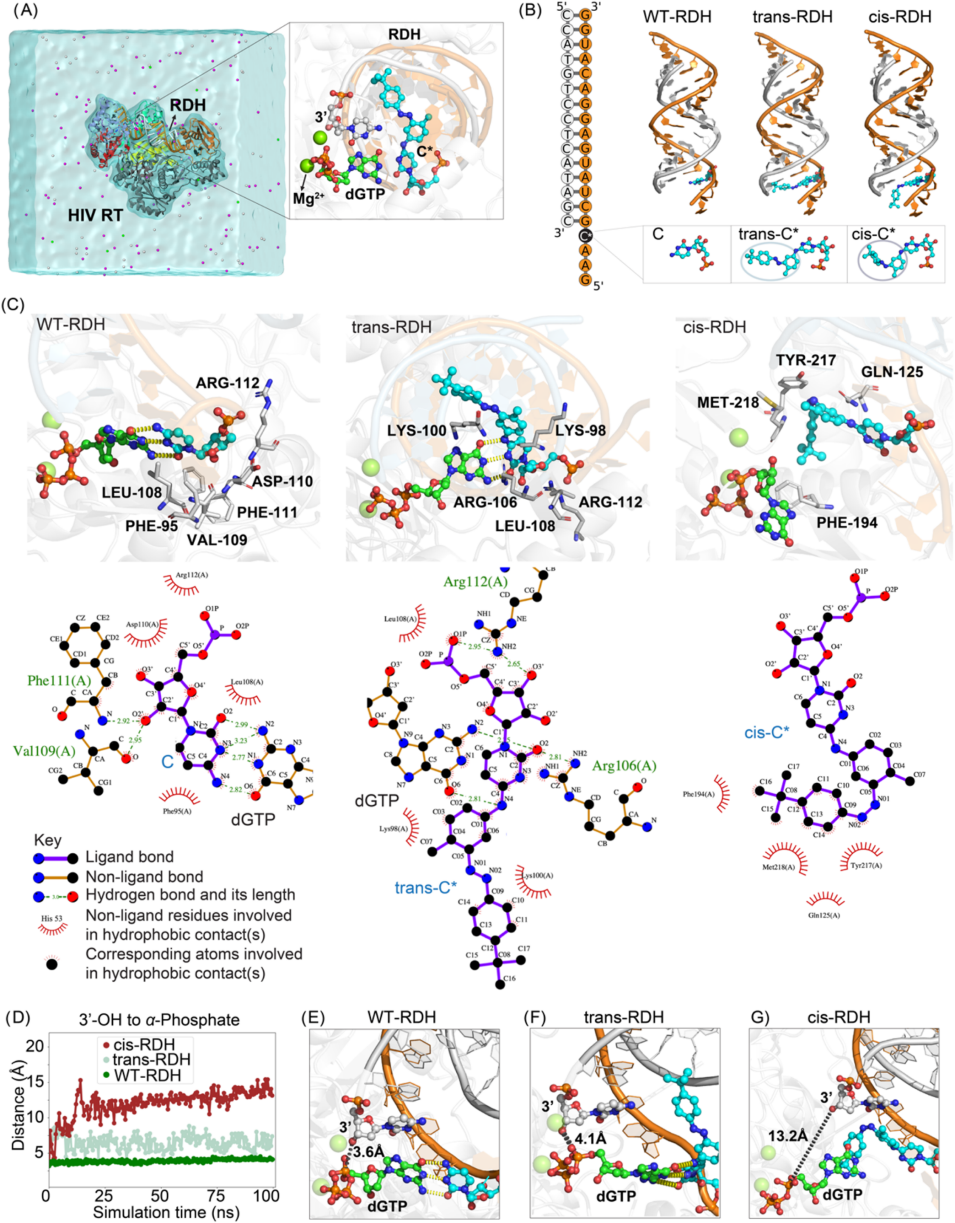
Structural analysis of the azobenzene modifications and optical control of reverse transcription in HIV RT. (A) Simulation setup of HIV RT enzyme with the 3′ DNA strand in the RNA:DNA hybrid (RDH). The inset details the positioning of dGTP-Mg^2+^ at the polymerase site. (B) Sequence and three-dimensional structure of the RDH duplex under study, the wild type (WT-RDH), azobenzene modification *trans*-RDH, and the modification after light exposure state *cis*-RDH. The RNA template is shown in orange and the complementary DNA primer in white. Insets below each sequence show the configurations of unmodified cytidine (C) and modified cytidine (*trans*-C* and *cis*-C*), respectively. (C) Interaction analysis of the templating cytidine, from the last frame of the MD simulation for WT-RDH, *trans-*RDH, and *cis-*RDH states. Enzyme residues involved in contact with the modified cytidine are highlighted in a stick representation. The bottom panels show a 2D map of the cytidine–enzyme interactions, generated by LigPlot.^44^ A similar analysis focusing on the incoming nucleotide is in Figures S9–S11. (D) The changes in the 3′-OH to dGTP-Mg^2+^ α-phosphate distance during simulation suggest the disruption of the catalytic distance at the cis state. (E–G) Last frame of the simulation is displayed focusing on the active site. The dotted black lines indicate 3′-OH to α-phosphate distance crucial for catalysis.

Similar to the 12mer RNA system (Figure 2), we observe changes in the structure of the biomolecular complex after modification (Figure 5C). The addition of the bulky *t*-butyl group induced changes in the positioning of the growing strand, resulting in alterations in the nearest contacts of the residue within the enzyme pocket. While the *trans*-C* remains at the enzyme pocket with a mostly altered network of interactions, the *cis*-C* state gets isolated within the enzyme pocket. Unlike WT and *trans*, in the light-induced *cis* state, the incoming nucleotide interacts with a completely different set of residues, such as Phe194, Gln125, Tyr217, and Met218. Despite differences in the network of interactions, the *trans* state retained the 3′-OH–dGTP-Mg^2+^ distance crucial for chemistry.^45^ In contrast, the light-activated *cis* state induces a drastic change, thereby hindering the proper alignment required for the nucleophilic attack (Figure 5D–G). Notably, we observe that the 3′-OH to α-phosphate distance responsible for chemistry increases from an ideal distance of 3.0 Å to 13.0 Å making the chemistry unlikely in the *cis* state. Overall, our simulations indicate that the *cis* modification positions dGTP-Mg^2+^ in a place that inhibits strand extension.

## Conclusions

We have designed and synthesized the azobenzene-modified cytidine phosphoramidite and successfully incorporated it into a series of oligoribonucleotides. The *E*–*Z* isomerization induced by different light sources was confirmed by the UV–vis spectrum, NMR, and HPLC in the context of both modified cytidine and oligonucleotide. The impact of the cytidine modification was studied through all-atom MD simulations and validated by UV melting experiments. We successfully conducted primer extension experiments with the azobenzene-modified cytidine phosphoramidite to assess the use of this modification to control biological function. We observe that the modification induces minimal changes to transcription prior to light activation. Upon light-induced activation of the azobenzene modification, a conformational transition happens at the active site that positions the incoming nucleotide to an inactive state where chemistry is no longer feasible. A limitation of this study is the potential cytotoxicity of UV light for *in vivo* applications, which future work will address by developing photoswitchable nucleotides that can be activated with lower-energy light sources. Overall, through light-induced excitation of the azobenzene-modified cytidine phosphoramidite, we successfully turn the transcription process on and off. This control over transcription is tested in various reverse transcriptase systems, confirming its general use. The potential application could be extended to other similar processes such as RNA replication, RNA cleavage, and RNA editing. This unique design enables reversible control of RNA’s biological functions in a minimally invasive way. The minimal structural disruption and efficient control of the biological function make this photoswitch system of great potential for the reversible optical control over biological processes involving oligonucleotides, such as siRNA, microRNA, aptamer, ribozyme, riboswitch, and other noncoding RNAs. Moreover, potential real-time regulation of enzymatic reactions using alternating light could be further explored in the future.

## Supplementary Material



## Notes

Additional experimental details, materials and methods, additional figures, characterization, and spectra (PDF)
