# Supplementary material for: Designing Reversible Photoswitching Azobenzene-Modified Nucleotide for Controlling Biological Function
Source: J Am Chem Soc. 2025 Jun 13;147(25):21638–48. doi: 10.1021/jacs.5c03252 (PMC12203576; doi:10.1021/jacs.5c03252)
Supplement: Supplementary file 1 [file ja5c03252_si_001.pdf]

Supporting Information

Designing reversible photoswitching azobenzene modified nucleotide for controlling biological function

Juncheng Li,†[1,2] Jinxi Du,†[1,2] Weiwei He,†[3,4] Ibrahim O. Adelakun,[1,2] Miao Zhong,[1,2] Savia Boyer,[1,2] Ya Ying Zheng,[1,2] Qishan Lin,[1] Serdal Kirmizialtin,*[,3,4] Jia Sheng,*[,1,2] and Ting Wang*[,1,2]

[1.]The RNA institute, University at Albany, State University of New York, Albany, New York, USA

[2.]Department of Chemistry, University at Albany, State University of New York, Albany, New York, USA

[3.]Chemistry Program, Science Division, New York University, Abu Dhabi, United Arab Emirates

[4.]Department of Chemistry, New York University, New York, USA

†These authors contributed equally to this work

Email: twang3@albany.edu
jsheng@albany.edu
serdal@nyu.edu

Table of Contents

General Experimental **3**

General Methods **3**

- **Synthesis of RNA Oligonucleotides** 3
- **High-Performance Liquid Chromatography (HPLC) Analysis and Purification** 4
- **Thermal Denaturation (T_m) Study** 4
- **Reverse Transcription (RT) Assays** 4
- **Initial Model Building** 5
- **Molecular Dynamics Simulations** 5
- **Quantum mechanical calculations** 6

Supporting Figures **7**

- **Synthetic Route to Obtain Compound 2.4** 7
- **HPLC Profile Analysis** 7
- **Interpreting Error Bars of Primer Extension Reactions** 8
- **Confirming effect of bulky groups on photoswitching functions** 10
- **Similar Analysis Focusing on the Incoming Nucleotide** 11

Photoswitch Experiments **13**

Experimental Procedures and Characterization Data **14**

References **19**

Orbitrap MS Analysis Spectrum **21**

Copies of Related NMR Spectra **24**

General Experimental

All commercially available chemicals were used without further purification unless otherwise noted. Reactions were monitored by thin-layer chromatography employing TLC silica gel 60-F254 plates. TLC plates were visualized by UV fluorescence (254 nm) or stained by Cerium Molybdate followed by heating. The reaction products were purified by column chromatography using Siliaflash-P60 (40-63 μm) silica gel available from Silicycle. ^{1}H-NMR spectra were recorded on a BRUKER AV-500 (500 MHz) and ^{13}C-NMR spectra were recorded on a BRUKER AV-500 (125 MHz). Data for ^{1}H-NMR are recorded as follows: chemical shift (δ, ppm), multiplicity (s = singlet, d = doublet, t = triplet, m = multiplet, q = quartet), coupling constant(s) in Hz and integration. Data for ^{13}C-NMR are reported in terms of chemical shift (δ, ppm). High-resolution mass spectra (HRMS) were obtained using Agilent Technologies 6530 Accurate Mass Q-TOF LC/MS for small molecules and Thermo Scientific LTQ Orbitrap Velos-negative mode for RNA sequences. High-performance liquid chromatography (HPLC) purification was implemented by Agilent 1100 system. Irradiation of photoswitch reactions was carried out using Thorlabs M365L3-365 nm Mounted LED, Thorlabs M385L3-385 nm Mounted LED, Thorlabs M405L4-405 nm Mounted LED, Thorlabs M430L5-430 nm Mounted LED, Thorlabs M455L4-455 nm Mounted LED, Thorlabs M505L4-505 nm Mounted LED with Thorlabs LEDD1B T-Cube LED Driver. UV-Vis spectra and melting temperature were obtained using an Agilent Cary 3500 Multicell UV-Vis Spectrophotometer Reverse transcription incubations were carried out using a Bio-Rad T100™ Thermal Cycler. Fluorescent gels were monitored using a Bio-Rad Universal Hood II Molecular Imager w/ CFW-1312M Camera.

General Methods

Synthesis of RNA Oligonucleotides

All oligonucleotides were chemically synthesized at a scale of 1.0 μmol using solid-phase synthesis utilizing the ASM Oligo-800 synthesizer. The azobenzene phosphoramidite was dissolved in acetonitrile to achieve a concentration of 0.1 M. I_2 (0.02 M) in a THF/Py/H_2O solution served as the oxidizing agent. Coupling was performed using a 0.25 M solution of 5-ethylthio-1H-tetrazole in acetonitrile for 12 minutes, applicable to both native and modified phosphoramidites. A 3 % solution of trichloroacetic acid in methylene chloride was employed for the 5′-detritylation. Synthesis was conducted on control-pore glass (CPG-1000) that was immobilized with the relevant nucleoside via a succinate linker. All reagents utilized are standard solutions procured from ChemGenes Corporation. The oligonucleotide was synthesized in the DMTr-off form. Following synthesis, the oligonucleotides were detached from the solid support and completely deprotected using a 1:1 v/v solution of ammonium hydroxide (28 % NH_3 in H_2O) and methylamine (40 % w/w aqueous solution) at 65 °C for 45 minutes. The solution was concentrated to dryness using a Speed-Vac concentrator. Subsequently, the solid was dissolved in 100 μL of DMSO and desilylated using a

triethylamine trihydrogen fluoride ($Et_3N{\bullet}3HF$) solution at 65 °C for 2.5 hours. Upon cooling to room temperature, the RNA was precipitated by the addition of 0.025 mL of 3 M sodium acetate and 1 mL of ethanol. The solution was cooled to -80 °C overnight prior to RNA recovery via centrifugation and finally dried over vacuum[1-4].

High-Performance Liquid Chromatography (HPLC) Analysis and Purification

RNA oligonucleotides were purified using HPLC on an XBridge Premier Oligonucleotide BEH C18 Column from Waters at a flow rate of 1 mL/min. Buffer A comprised 0.1 M triethylammonium acetate (pH 7.0), while buffer B consisted of 100 % acetonitrile. The RNA oligonucleotides were eluted using a linear gradient of 5–45 % buffer B over 15 minutes. The obtained fractions were lyophilized, desalted using Waters Sep-Pac C18 columns, and subsequently re-concentrated.

Thermal Denaturation (T_m) Study

Duplex RNA solutions (1.5 μM) were prepared by mixing foil-wrapped purified *cis* and *trans* RNA from HPLC with corresponding RNA strands in a sodium phosphate buffer (10 mM, pH 7.0) containing 100 mM NaCl. The solutions were heated to 95 °C for 5 minutes, subsequently cooled gradually to room temperature, and stored at 4 °C for 2 hours before T_m measurement. Thermal denaturation was conducted using a Cary 300 UV–Visible Spectrophotometer equipped with a temperature controller. The temperature reported is the block temperature. The denaturizing curve was obtained at 260 nm by heating and cooling from 10 to 80 °C four times at a rate of 0.5 °C/min. All melting curves were replicated four times. The thermodynamic parameters of each strand were derived by fitting the melting curves using Meltwin software[5-7].

Reverse Transcription (RT) Assays

Reverse transcription assays were conducted using AMV reverse transcriptase (New England Biolabs), HIV-1 reverse transcriptase (MilliporeSigma), MMLV reverse transcriptase (Promega), and MultiScribe reverse transcriptase (Invitrogen) in a total volume of 20 μL, comprising 10× reverse transcription buffer: 50 mM Tris (pH 8.3), 75 mM KCl, 3 mM $MgCl_2$, and 10 mM DTT. The final reaction mixes comprised an RNA template (3 μM), a DNA FAM-primer (1.5 μM), and dNTP (1 mM). *Trans*-RNA templates were used under dark without any pre-activation, while *cis*-RNA templates were obtained by pre-activation by 365 nm LED lights for 1 hour. Following the incorporation of each reverse transcriptase, AMV RT (1 U), HIV-1 RT (0.5 U), M-MLV (100 U), and MultiScribe (50 U), the mixtures were incubated at 37 °C for 1 hour. The reactions were quenched using a stop solution composed of 98% formamide, 0.05% xylene cyanol, and 0.05% bromophenol blue, thereafter, heated to 95 °C for 5 minutes, and then cooled to 0 °C in an ice bath. The reactions were analyzed using 15% PAGE 8 M urea at 250 V for 1 hour. Fluorescent and UV gel imaging was conducted using a Bio-Rad Gel XRS+ imager[4].

Initial Model Building

The wild-type 12-base-paired RNA duplex (WT-12mer) with the sequence 5′-AAUGCCGCACUG-3′ and its complementary strand was constructed using the Nucleic Acid Builder (NAB)[8] program in A-form geometry, a standard conformation for RNA. To investigate the effects of azobenzene modifications, *trans* and *cis* RNA variants were prepared by substituting C6 in the wild-type structure with modified bases using PyMOL [Schrödinger, LLC], generating *trans*-12mer and *cis*-12mer. MD simulations were conducted for WT-12mer, *trans*-12mer, and *cis*-12mer to evaluate the impact on RNA duplex stability. Additionally, two modified *cis*-12mer variants, namely *cis*-12mer-t (*cis*-12mer with the *tert*-butyl group removed) and *cis*-12mer-m (*cis*-12mer with the *ortho*-methyl group removed), were modeled under the same conditions to isolate the contribution of each bulky substituent.

A high-resolution (3.2 A) X-ray crystal structure corresponding to close form of the HIV-RT enzyme (PDB ID: 1RTD)[9] was used to build initial structures for MD simulations of RT-related systems. Additionally, the wild-type RNA:DNA hybrid (RDH) duplex, referred to as WT-RDH, was constructed by combining RNA and DNA strands with complementary sequences, generated in A-form using the NAB[8] program. The RNA template strand has the 19-nt sequence 5′-GAACGCUAUGAGGACAUGG-3′, while the 15-nt DNA primer strand is 5′-CCATGTCCTCATAGC-3′. The initial structures of the trans and cis variants were generated using PyMOL [Schrödinger, LLC] by substituting C4 of the RNA strand in the wild-type structure with the corresponding modification, herein referred to as trans-RDH and cis-RDH, respectively. The deoxyguanosine triphosphate (dGTP) model was prepared using the tLEaP module of AMBER22 program[10]. The dGTP molecule was positioned in rough alignment with the incoming nucleotide (*i.e.* TTP) in the closed-form complex[9], and the RDH duplex was aligned relative to the primer-template DNA of the crystal structure to simulate the process of dGTP incorporation into the growing strand during reverse transcription. MD simulations of the HIV RT primer–template complex was conducted for WT-RDH, *trans*-RDH, and *cis*-RDH, respectively.

Molecular Dynamics Simulations

All-atom MD simulations were employed to investigate the structure and dynamics of azobenzene modified systems, including 12mer RNA duplex and the RDH duplex conjugated with HIV-RT enzyme. All of the MD simulations were carried out using the GROMACS 2018.8[11] suit of programs. The HB-CUFIX force field, including base-stacking and hydrogen-bonding corrections for RNA[12] and Amber14SB parameters for proteins[13, 14] was used to describe the protein and the nucleic acid interactions. We used the TIP3P[15] model for water, and NBFIX[16] parameters for ions. Parameters for the modified bases were derived and supplemented from the Generalized Amber Force Field (GAFF)[17], with partial atomic charges calculated using RED.v.III.52 via RESP-A1 scheme[18] . A soft restraint with a force constant of 500 kJ mol^{-1} nm^{-2} was applied to the azobenzene group to maintain its trans or cis configuration throughout the simulations. dGTP parameters were obtained using the same established approach[17-20] and the active-site magnesium ions were modelled via point

charge[21, 22]. In all simulations, the total charge was neutralized with counter ions, and the biomolecules were solvated in an explicit triclinic water box, with at least 25.0 Å between the box edge and solute in each dimension. In all simulations, a leap-frog[23] integrator was used with a time step of 2 fs. To maintain the covalent bonds and water geometry, we employed the LINCS[24] and SETTLE[25] algorithms respectively. We used the particle mesh Ewald (PME)[26] scheme to account for the electrostatic interactions using a real space-cutoff of 1.1 nm. Periodic boundary conditions were applied in all directions throughout all steps.

Simulations of all 12mer RNA duplexes were conducted in a 110 mM Na^+ solution to replicate experimental conditions. Sodium and chloride ions were added to neutralize the system and achieve the target ionic strength. The solvated system underwent a 5000-step energy minimization using the steepest descent algorithm to optimize initial configurations and resolve issues arising from the random placement of water molecules and ions. Subsequent equilibration, addressing both volume and solvent, was performed in multiple stages. Initially, a short 2.5 ns simulation in the isothermal–isobaric ensemble (NPT) at 300 K and 1 bar was carried out to determine the box volume, using a velocity-scaling thermostat[27] and a Parrinello–Rahman barostat[28]. Following this, a 100 ns simulation in the canonical (NVT) ensemble was conducted with harmonic positional restraints applied to the RNA duplex, allowing the solvent and ions to equilibrate freely. Finally, production runs were performed for 1 μs in the NVT ensemble without restraints, with RNA coordinates recorded every 5 ps for subsequent analysis.

For HIV RT systems, magnesium, potassium, and chloride ions were added to ensure neutrality, mimicking the experimental conditions with a 40 mM K^+ / 1.5 mM Mg^{2+} concentration. The solvated configurations underwent 5000-step energy minimization via steepest descent scheme to refine the bad contacts due to the random insertion of water and ions. Subsequently, a multi-step equilibration was performed as follows: a 2.5 ns simulation at 300 K and 1 bar in the NPT ensemble using a velocity-scaling thermostat[27] and Parrinello-Rahman[28], followed by a 10 ns-long NVT ensemble simulation, where the enzyme−RDH-dGTP complex was frozen, allowing only solution atoms to move. The system was further equilibrated for 10 ns to relax the RDH duplex and establish enzyme-RDH interactions, followed by a 5 ns equilibration to adjust the dGTP position, ensuring its α-phosphate was within 3 Å of the 3′-OH group in the primer strand[29]. Finally, all systems were simulated for 100 ns of unbiased MD in the NVT ensemble, with data recorded every 5 ps, and the resulting trajectories used for further analysis.

Quantum mechanical calculations

To assess changes in electronic properties induced by bulky substituents, we calculated the HOMO–LUMO levels of a series of *trans*-azobenzene–modified cytosine derivatives with systematic removal of these groups: 1) our current design, 2) (*E*)-2′-methylazobenzene, 3) (*E*)-4-*tert*-butylazobenzene, and 4) unsubstituted (*E*)-azobenzene. All electronic structure calculations were performed at the B3LYP/6-311G(d) level using the Gaussian16 programme[30], and the molecular orbital profiles are visualized and rendered by using Visual Molecular Dynamics (VMD) tool[31].

Supporting Figures

Synthetic Route to Obtain Compound 2.4

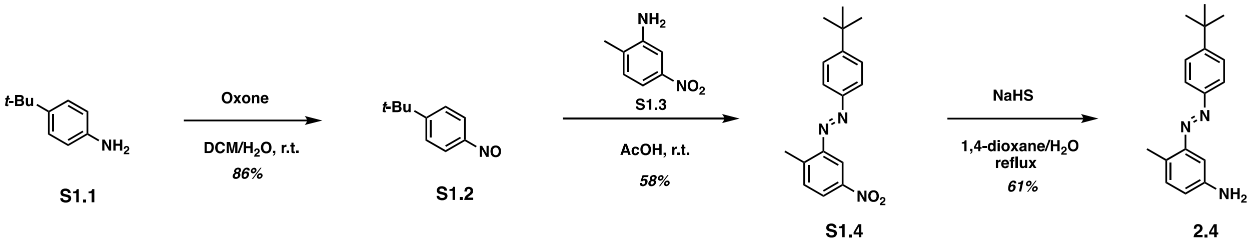

Figure S1. Synthesis of compound 2.4. The synthetic route starts from oxidization of a commercially available 4-*tert*-butylaniline S1.1 to afford the corresponding nitroso benzene S1.2 with 86% yield. The azobenzene fragment S1.4 was yielded by a Baeyer-Mills reaction between S1.2 and an aniline S1.3 with 58% yield. The optimal condition of specifically reducing the nitrobenzene is to use sodium hydrosulfide in 1,4-dioxane/water (1:1) to obtain 2.4 without over-reducing the diazene.

HPLC Profile Analysis

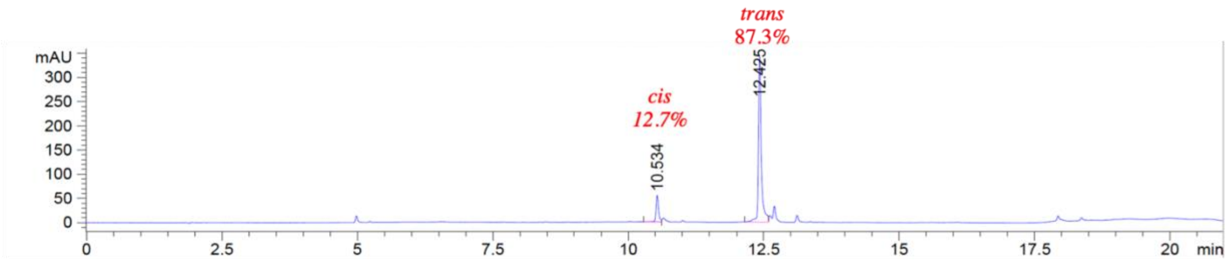

Figure S2. HPLC profile of fresh synthesized modified RNA sequence 5′ -AAUGCC*GCACUG-3′. After purifying by using PAGE gel, we ran the HPLC for pure RNA strand 1 (5′ -AAUGCC*GCACUG-3′). Two distinct peaks were observed, corresponding to the 87.3% *trans* and 12.7% *cis* isomers, respectively.

Interpreting Error Bars of Primer Extension Reactions

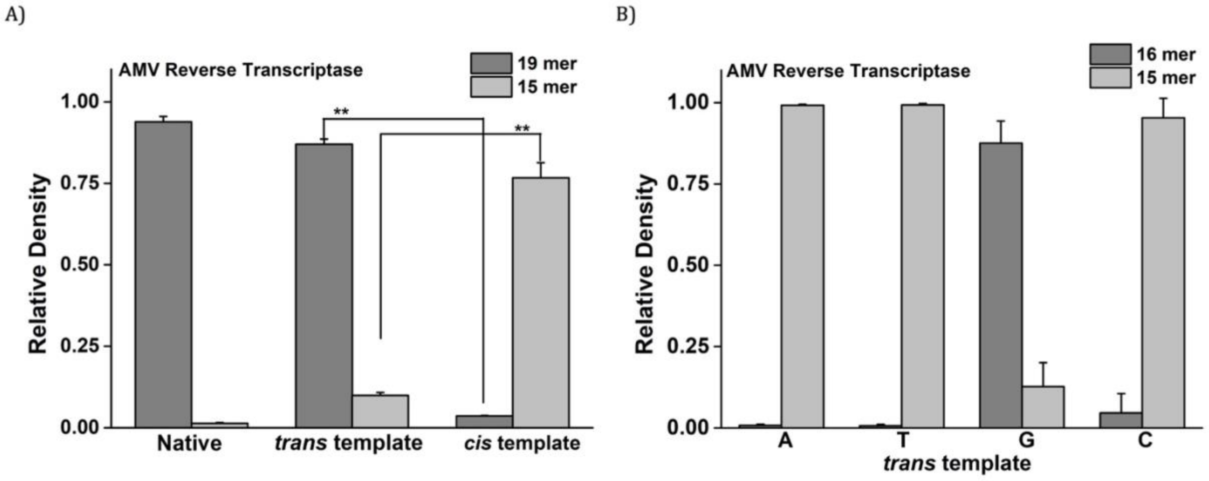

Figure S3. Mean ± SE from triplicates of AMV promoted reverse transcription. * represents 0.01< *P* < *0.05,* ** represents *P* < 0.01. A) Fluorescent Gel behavior (15mer→19mer) of native template *vs. trans* template *vs. cis* template in the presence of dNTP. B) Fluorescent Gel behavior (15mer→16mer) of the *trans* template incorporated with dATP, dTTP, dGTP and dCTP, respectively.

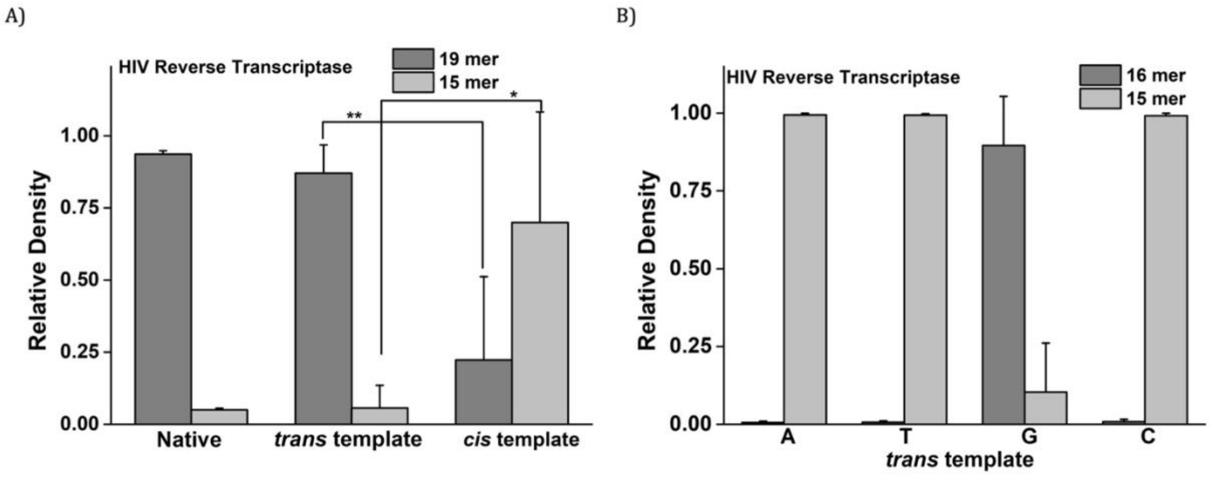

Figure S4. Mean ± SE from triplicates of HIV promoted reverse transcription. * represents 0.01< *P* < *0.05,* ** represents *P* < 0.01. A) Fluorescent Gel behavior (15mer→19mer) of native template *vs. trans* template *vs. cis* template in the presence of dNTP. B) Fluorescent Gel behavior (15mer→16mer) of the *trans* template incorporated with dATP, dTTP, dGTP and dCTP, respectively.

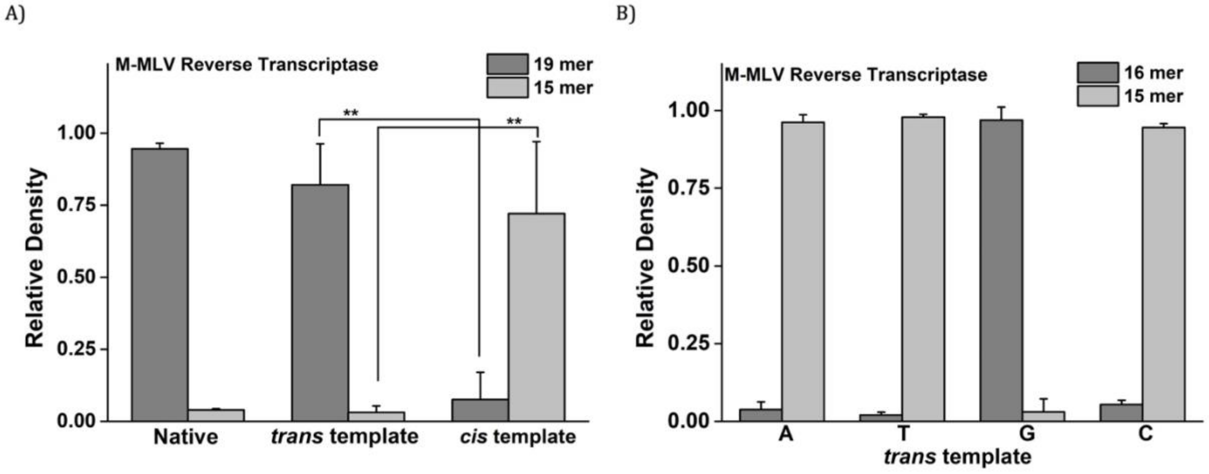

Figure S5. Mean ± SE from triplicates of M-MLV promoted reverse transcription. * represents 0.01< *P* < *0.05,* ** represents *P* < 0.01. A) Fluorescent Gel behavior (15mer→19mer) of native template *vs. trans* template *vs. cis* template in the presence of dNTP. B) Fluorescent Gel behavior (15mer→16mer) of the *trans* template incorporated with dATP, dTTP, dGTP and dCTP, respectively.

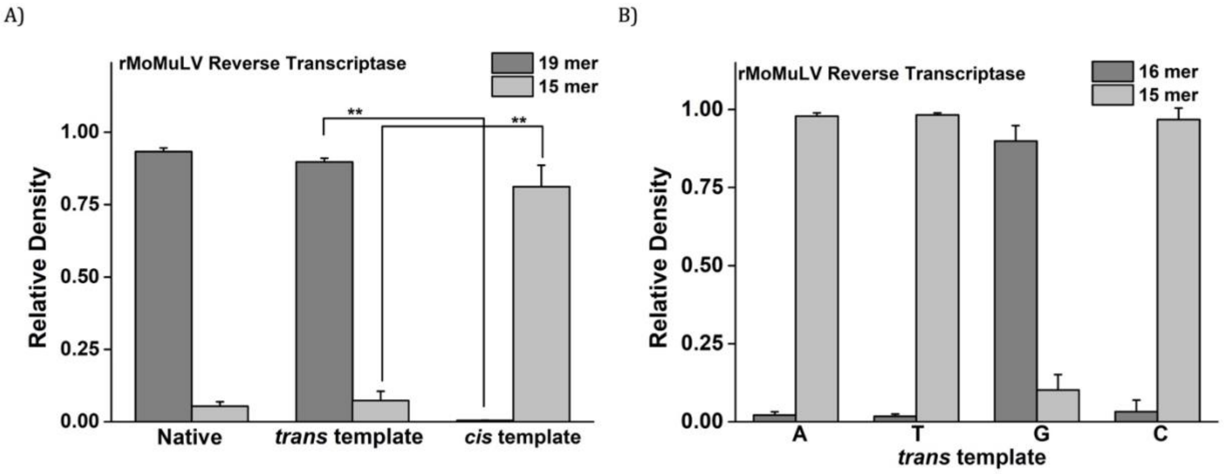

Figure S6. Mean ± SE from triplicates of MultiScribe (rMoMuLV) promoted reverse transcription. * represents 0.01< *P* < *0.05,* ** represents *P* < 0.01. A) Fluorescent Gel behavior (15mer→19mer) of native template *vs. trans* template *vs. cis* template in the presence of dNTP. B) Fluorescent Gel behavior (15mer→16mer) of the *trans* template incorporated with dATP, dTTP, dGTP and dCTP, respectively.

Confirming effect of bulky groups on photoswitching functions

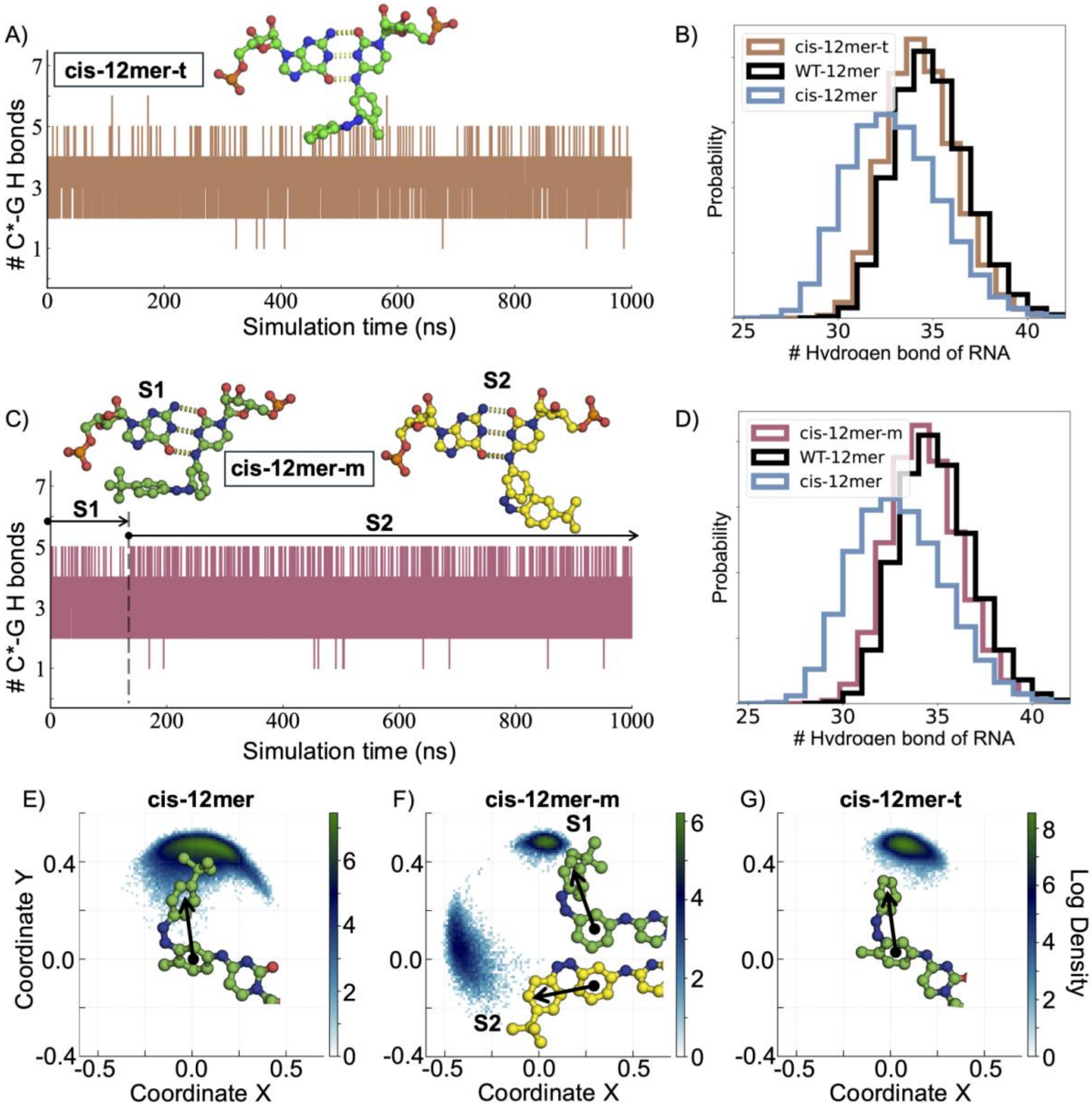

Figure S7. Comparison of *cis*-12mer derivatives lacking bulky groups with current cis-12mer. A–B) *cis*-12mer-t: *cis*-12mer with the *tert*-butyl group removed. A) Time evolution of hydrogen bonds between modified cytidine (C*) and its complementary guanine; B) probability distribution of total hydrogen bonds across the RNA duplex. C–D) *cis*-12mer-m: *cis*-12mer with the *ortho*-methyl group removed. C) Time evolution of hydrogen bonds between C* and guanine, and the dynamics of the modified cytidine are partitioned into two states: *tert*-butyl inward (S1) and *tert*-butyl outward (S2). Insets show representative structures of S1 and S2 configurations; D) probability distribution of total hydrogen bonds in the RNA duplex. E–F) Orientation distribution of the vector connecting the two benzene rings of the azobenzene moiety evaluated from MD simulations of E) *cis*-12mer, F) *cis*-12mer-m, and G) *cis*-12mer-t.

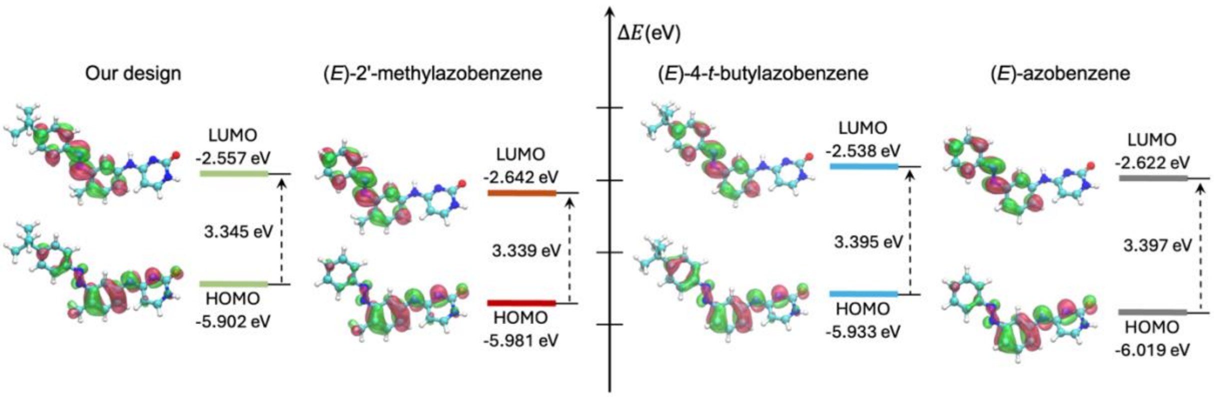

Figure S8. HOMO–LUMO energy diagrams of *trans*-azobenzene-modified cytosine derivatives for comparison. From left to right: the current design, with the *tert*-butyl group removed, with the *ortho*-methyl group removed, and with both bulky groups removed.

Similar Analysis Focusing on the Incoming Nucleotide

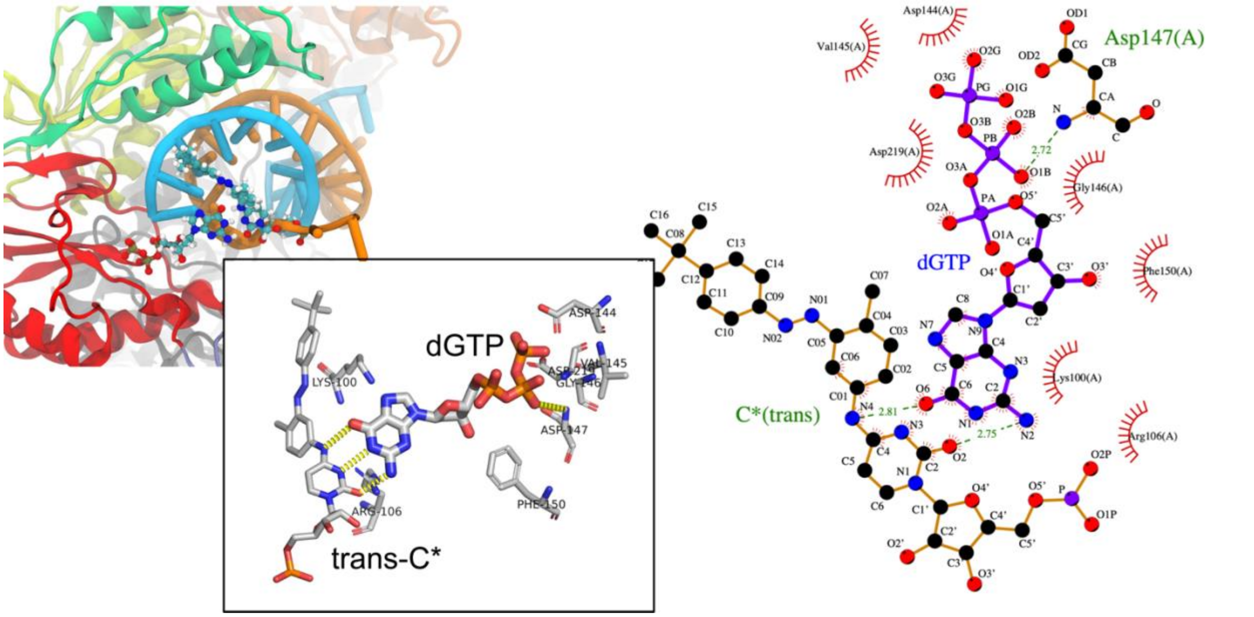

Figure S9. LigPlus analysis illustrating the interaction between dGTP and the neighboring residues *trans*-RDH system.

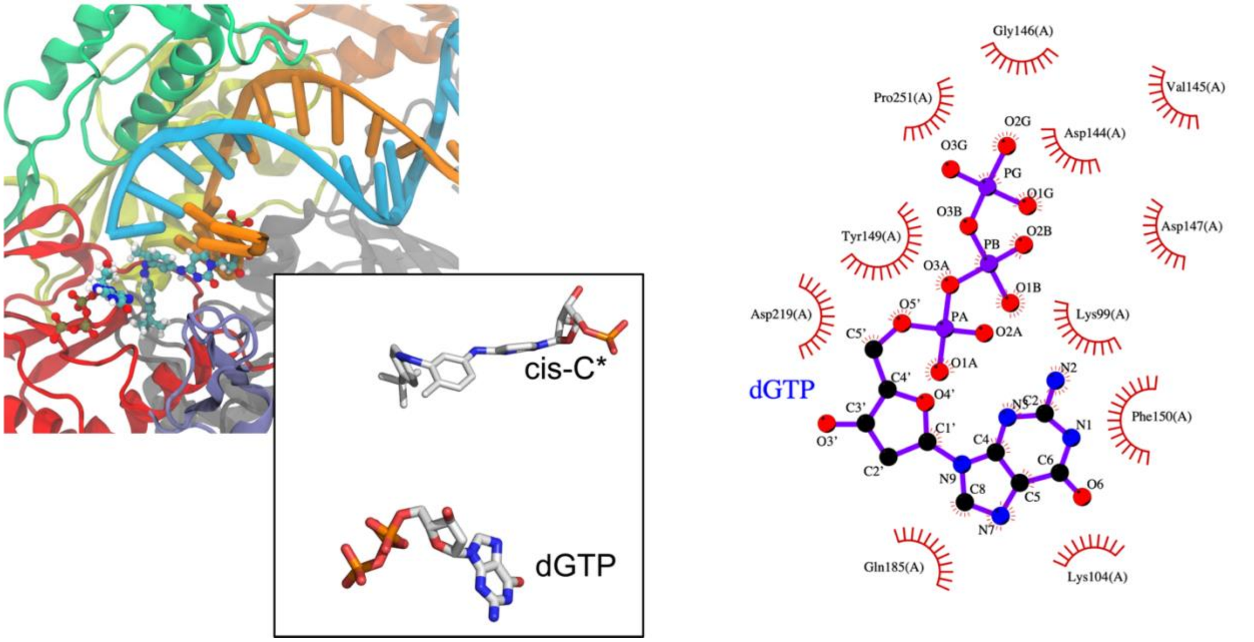

Figure S10. LigPlus analysis illustrating the interaction between dGTP and the neighboring residues *cis*-RDH system.

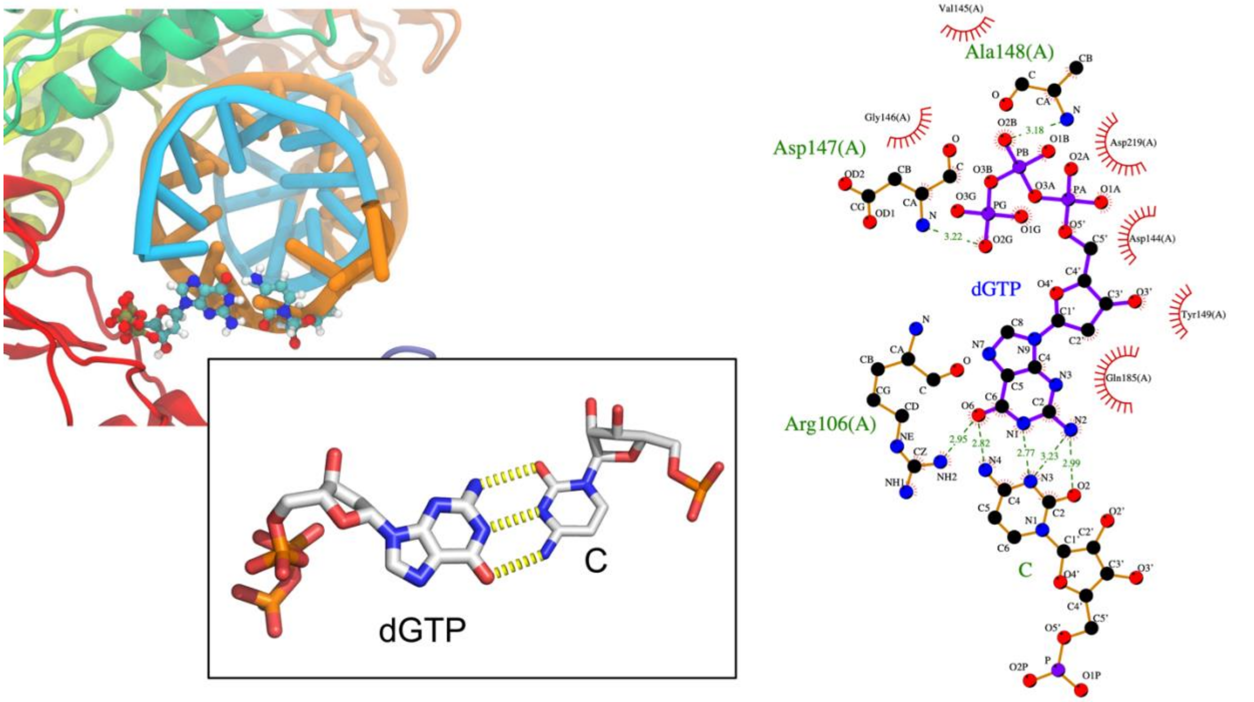

Figure S11. LigPlus analysis illustrating the interaction between dGTP and the neighboring residues WT-RDH system.

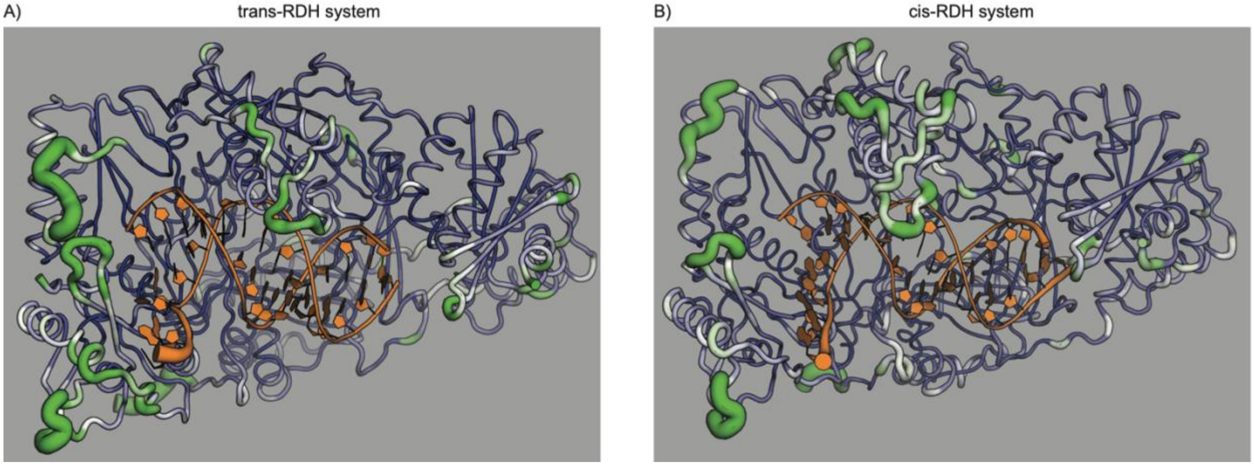

Figure S12. Thermal parameter (B-factor) distribution in the HIV RT enzyme is presented with dGTP hidden to highlight the conformational changes in the enzyme: A) *trans*-RDH and B) *cis*-RDH, both displayed using a "putty" representation, as implemented in PyMOL.

Photoswitch Experiments

Irradiation of isomerization were carried out using 880 mW M365L3 365 nm UV mounted LED and 900 mW M455L3 455 nm mounted Blue LED. The conversion yields were quantified by 500 M Hydrogen NMR. The reaction was running with 5 mM concentration in DMSO at room temperature. The concentration for UV scan is consistent in 50 µM in DMSO.

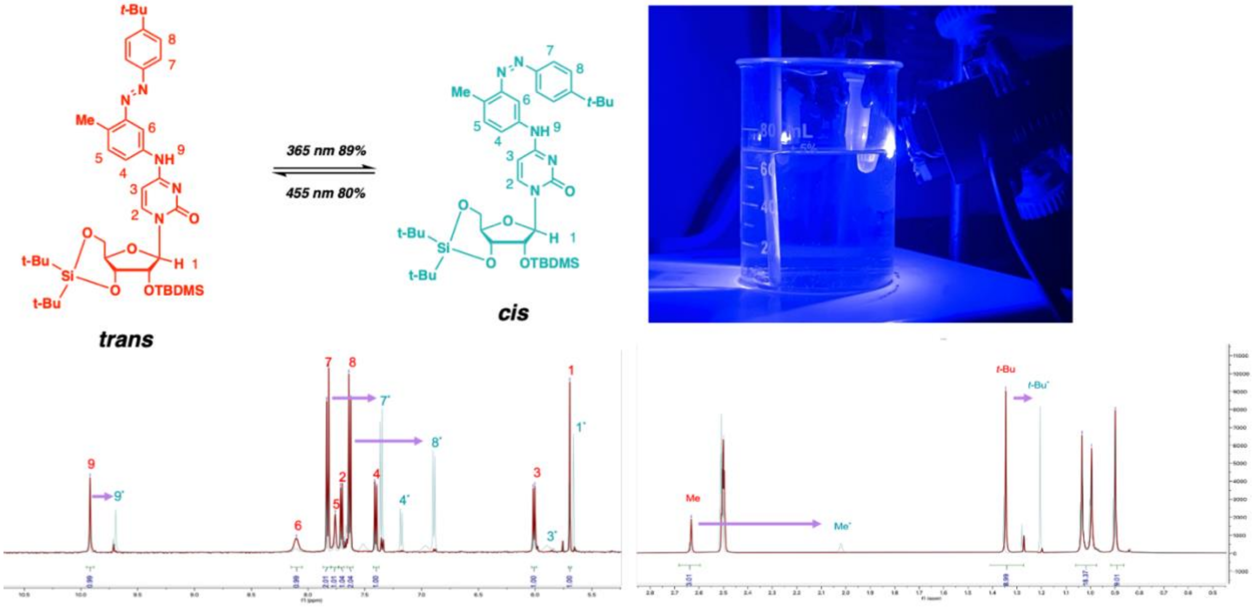

50 mg molecule was dissolved in 0.8 mL DMSO-d_6 and then characterized by 500M Hydrogen NMR. To quantify the conversion yield, the sample in DMSO-d_6 was irradiated under different LED lights for 20 mins and recharacterized by NMR.

Experimental Procedures and Characterization Data

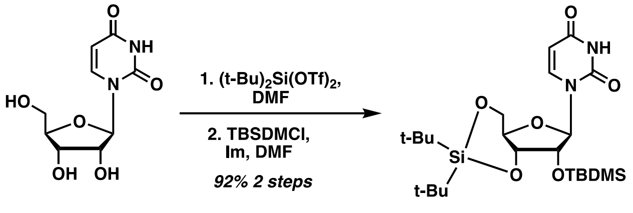

2.1 **2.2**

Uridine **2.1** (2 g, 8.2 mmol, 1.0 eq) was dissolved in dry DMF (10 mL) and the solution was stirred at 0 °C. (t-Bu)$_2$(OTf)$_2$Si (4.4 g, 9.9 mmol, 1.2 eq) was added dropwise over 30 mins. After another 30 mins stirring at 0°C, imidazole (2.8 g, 41 mmol, 5 eq) was added and the reaction was allowed to warm to room temperature over a period of 30 mins. Then, *tert*-butyldimethylchlorosilane (1.5 g, 9.9 mmol, 1.2 eq) was added portion-wise and the reaction was heated to 60 °C for 4 h. Subsequently, the reaction mixture was diluted with EtOAc (200 mL) and extracted twice with $NaHCO_3$ (200 mL) and water (200 mL). The organic layer was dried over Na_2SO_4 and the solvent was removed by rotatory evaporator. The crude product was purified by chromatography (Hexane:EtOAc = 5:1), giving the product as a white solid (92 %, 3.7 g).

^{1}H NMR (500 MHz, $CDCl_3$) δ 8.85 (s, 1H), 7.26-7.24 (d, J = 7.9 Hz, 1H), 5.75-5.74 (d, J = 8.2 Hz, 1H), 5.67 (s, 1H), 4.52-4.49 (dd, J = 9.4, 5.1 Hz, 1H), 4.29-4.28 (d, J = 4.4 Hz, 1H), 4.18-4.14 (m, 1H), 3.99-3.95 (t, J = 10.0 Hz, 1H), 3.88-3.85 (dd, J = 9.7, 4.7 Hz, 1H), 1.05 (s, 9H), 1.02 (s, 9H), 0.93 (s, 9H), 0.18 (s, 3H), 0.14 (s, 3H).

^{13}C NMR (126 MHz, $CDCl_3$) δ 163.97, 149.97, 139.53, 102.35, 93.97, 76.00, 75.30, 74.51, 67.54, 27.46, 26.97, 25.84, 22.74, 20.31, 18.22, -4.32, -5.02.

HRMS(ESI$^+$) calculated for $C_{23}H_{42}N_2O_6Si_2$, [MH$^+$]: 499.2654 (calcd), 499.2776(found).

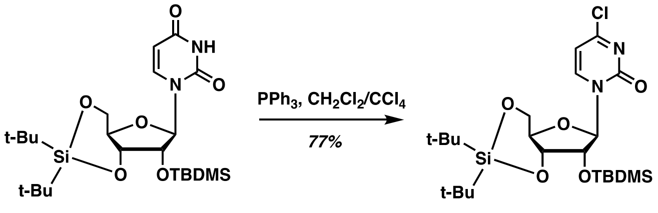

2.2 **2.3**

A solution of silyl-protected uridine (2.47 mmol, 1.23 g, 1.0 eq) and triphenylphosphine (6.17 mmol, 1.61 g, 2.5 eq) in a mixture of DCM (20 mL) and CCl_4 (20 mL) was refluxed for 2.5 h. After completion of reaction, the solvent was removed by rotatory evaporator. The crude product was purified by chromatography (Hexane:EtOAc = 10:1) to yield pale yellow powder with 77 % yield.

^{1}H NMR (500 MHz, $CDCl_3$) δ 7.75-7.73 (d, J = 7.2 Hz, 1H), 6.39-6.38 (d, J = 7.0 Hz, 1H), 5.63 (s, 1H), 4.52-4.49 (dd, J = 9.3, 5.3 Hz, 1H), 4.29-4.28 (d, J = 4.3 Hz, 1H), 4.27-4.24 (dd, J = 10.1, 5.2 Hz, 1H), 3.98 – 3.95 (m, 1H), 3.73-3.70 (dd, J = 9.8, 4.3 Hz, 1H), 0.98 (s, 18H), 0.91 (s, 9H), 0.21 (s, 3H), 0.13 (s, 3H).

^{13}C NMR (126 MHz, $CDCl_3$) δ 166.76, 152.79, 142.91, 105.18, 94.53, 75.62, 75.10, 74.92, 67.66, 27.44, 26.93, 25.89, 22.73, 20.31, 18.15, -4.27, -4.90.

HRMS(ESI$^+$) calculated for $C_{23}H_{41}ClN_2O_5Si_2$, [MH$^+$]: 517.2315 (calcd), 517.2445(found).

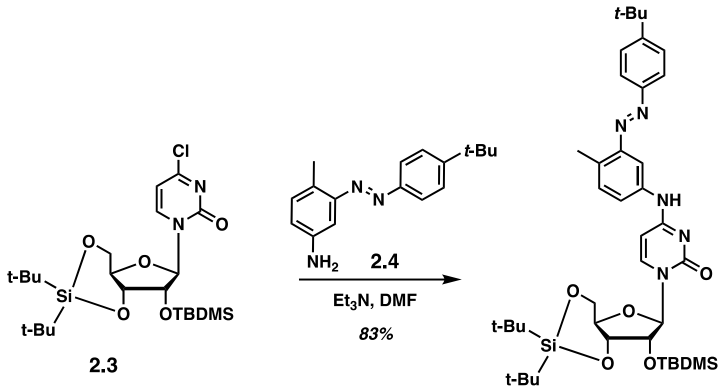

2.3 2.5

A mixture of silyl-protected chloride-substituted cytidine (1.58 g, 3 mmol, 1.0 eq), azobenzene-aniline (818 mg, 3 mmol, 1.0 eq) in DMF (25 mL) was stirred overnight at room temperature. The reaction mixture was quenched by H_2O (50 mL) when the TLC monitoring (MeOH:DCM = 1:10) showed the completion of the coupling reaction. The orange suspended solid was filtered by Büchner funnel. Then, the orange powder was redissolved in 50mL DCM and followed by washing with H_2O (50 mL×3). The organic layers were combined and dried over anhydrous Na_2SO_4. Then the solvent was evaporated with reduced pressure to give the crude product. The residue was purified by silica gel chromatography (3 % MeOH in DCM) to afford 1.32 g orange solid with 83 % yield.

^{1}H NMR (500 MHz, DMSO) δ 9.92 (s, 1H), 8.10 (s, 1H), 7.83 (d, *J* = 8.7 Hz, 2H), 7.76 (s, 1H), 7.70 (d, *J* = 7.5 Hz, 1H), 7.63 (d, *J* = 8.7 Hz, 2H), 7.40 (d, *J* = 8.5 Hz, 1H), 6.01 (d, *J* = 7.5 Hz, 1H), 5.69 (s, 1H), 4.40 (dd, *J* = 9.1, 5.0 Hz, 1H), 4.33 (d, *J* = 4.8 Hz, 1H), 4.10 – 4.01 (m, 2H), 3.97 (dd, *J* = 10.0, 5.0 Hz, 1H), 2.63 (s, 3H), 1.35 (s, 9H), 1.03 (s, 9H), 1.00 (s, 9H), 0.90 (s, 9H), 0.16 (s, 3H), 0.10 (s, 3H).

^{13}C NMR (126 MHz, $CDCl_3$) δ 164.58, 154.86, 154.05, 150.71, 143.10, 139.66, 137.81, 132.31, 130.99, 126.04, 122.86, 115.97, 98.40, 94.62, 94.25, 75.83, 75.31, 74.64, 67.76, 35.05, 31.26, 27.52, 26.97, 25.90, 22.78, 20.34, 18.19, 17.12, -4.30, -4.83.

HRMS(ESI$^+$) calculated for $C_{40}H_{61}N_5O_5Si_2$ [MH^+]: 748.4284 (calcd), 748.4341 (found).

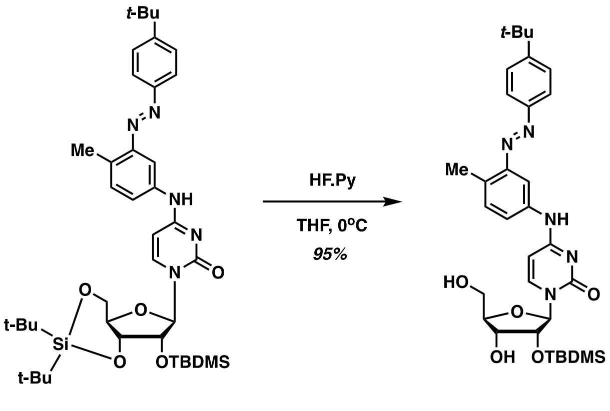

2.5 2.6

A mixture of azobenzene-modified cytidine (1.1 g, 1.47 mmol) in 60 mL THF was cooled to 0 °C in an ice bath and added a solution of hydrogen fluoride-pyridine complex (70 % HF, 30 % pyridine, 0.5 mL) in 3.75 mL pyridine. After 1h at 0 °C when the TLC monitoring

(MeOH:DCM = 1:10) indicated the completion of reaction, 10mL pyridine was added into the reaction mixture. The solution was extracted by DCM (50 mL×3) and the solvent was removed by rotary evaporator. The rest small amount pyridine was blow-dried by air flow until the pyridine was completely removed. Then, the residue was purified by silica gel chromatography (2 % MeOH in DCM) to give 849 mg product as orange powder with 95 % yield.

^{1}H NMR (500 MHz, $CDCl_3$) δ 8.94 (s, 1H), 7.78 (d, *J* = 8.4 Hz, 2H), 7.72 (s, 1H), 7.46 (d, *J* = 8.4 Hz, 2H), 7.23 (s, 1H), 7.17 (s, 1H), 6.83 (d, *J* = 8.5 Hz, 1H), 6.04 (s, 1H), 5.51 (s, 1H), 4.67 (t, *J* = 6.2 Hz, 1H), 4.22 (t, *J* = 4.8 Hz, 1H), 4.12 – 4.07 (m, 1H), 3.97 – 3.89 (m, 1H), 3.77 (d, *J* = 10.5 Hz, 1H), 3.01 (s, 1H), 2.59 (s, 3H), 1.33 (s, 9H), 0.84 (s, 9H), 0.05 (s, 3H), 0.04 (s, 3H).

^{13}C NMR (126 MHz, $CDCl_3$) δ 156.12, 154.60, 153.81, 151.79, 150.74, 150.27, 131.72, 125.96, 125.65, 122.82, 121.35, 94.99, 94.55, 85.50, 85.36, 74.34, 74.10, 70.27, 61.56, 34.99, 31.27, 25.75, 17.99, 17.04, -4.67, -5.13.

HRMS(ESI$^+$) calculated for $C_{32}H_{45}N_5O_5Si$ [MH$^+$]: 608.3263 (calcd), 608.3334 (found).

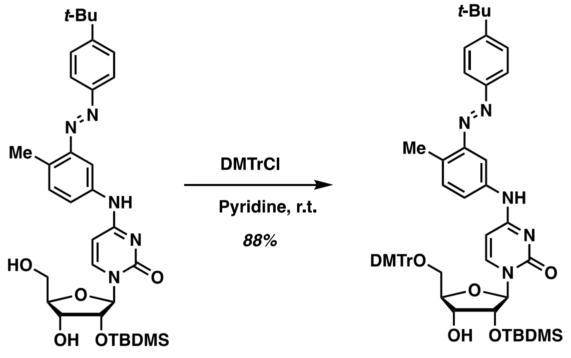

2.6 **2.7**

To a solution of compound pentaglucose (826 mg, 1.36 mmol, 1.0 eq) in dry pyridine (10 mL) was added 4,4′-Dimethoxytrityl chloride (DMTrCl, 920 mg, 2.72 mmol, 2.0 eq) under dark. The resulting solution was stirred overnight at room temperature under dark. The reaction was quenched with methanol (2 mL) and stirred for another 5 mins. After concentration under vacuum, the residue was purified by silica gel chromatography (5 % MeOH in dichloromethane) to give 684 mg product as orange powder with 88 % yield.

^{1}H NMR (500 MHz, CD_2Cl_2) δ 8.57 (d, *J* = 4.2 Hz, 1H), 8.10 (d, *J* = 7.6 Hz, 1H), 7.84 (d, *J* = 8.7 Hz, 2H), 7.59 (s, 1H), 7.54 (d, *J* = 8.6 Hz, 2H), 7.35 – 7.25 (m, 8H), 7.13 (d, *J* = 7.8 Hz, 1H), 6.90 – 6.80 (m, 5H), 5.93 (s, 1H), 5.65 (d, *J* = 7.6 Hz, 1H), 4.35 – 4.24 (m, 2H), 4.07 (d, *J* = 7.3 Hz, 1H), 3.74 (s, 3H), 3.73 (s, 3H), 3.55 (dd, *J* = 11.1, 2.3 Hz, 1H), 3.47 (dd, *J* = 11.1, 3.0 Hz, 1H), 2.71 (s, 3H), 1.38 (s, 9H), 0.94 (s, 9H), 0.28 (s, 3H), 0.18 (s, 3H).

^{13}C NMR (126 MHz, CD_2Cl_2) δ 158.82, 158.77, 158.73, 158.70, 155.32, 154.78, 150.82, 149.71, 147.65, 144.86, 139.63, 135.93, 135.43, 135.30, 131.79, 130.24, 130.10, 129.09, 128.09, 127.91, 127.76, 127.73, 126.95, 126.88, 126.05, 125.55, 123.74, 122.73, 121.02, 113.21, 113.05, 90.34, 86.87, 82.81, 81.19, 76.69, 69.47, 61.85, 55.21, 55.18, 34.91, 31.01, 25.63, 17.99, 16.81, -4.62, -5.57.

HRMS(ESI$^+$) calculated for $C_{53}H_{63}N_5O_7Si$ [MH$^+$]: 910.4570 (calcd), 910.4594 (found).

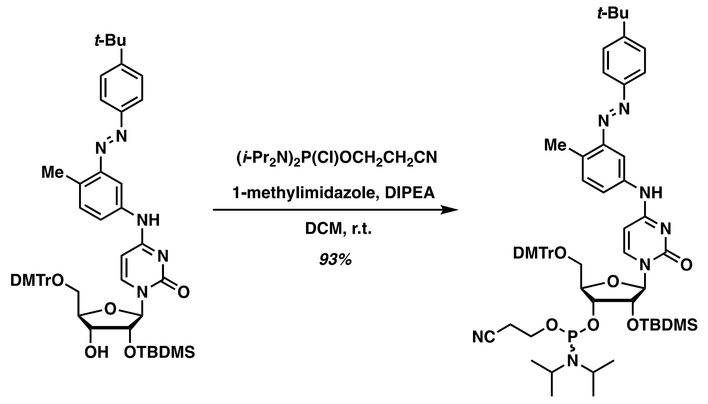

2.7 3.1

To a solution of starting material (414 mg, 0.45 mmol, 1.0 eq) in DCM was added DIPEA (290 mg, 2.25 mmol, 5.0 eq), 1-methylimidazole (37 mg, 0.45 mmol, 1.0 eq) and 2-cyanoethyl *N,N*-diisopropylchlorophosphoramidite (319 mg, 1.35 mmol, 3.0 eq). The resulting solution was stirred overnight at room temperature under Ar. Then, the reaction was washed with H_2O for at least 3 times and extracted with DCM. After drying the organic layer over Na_2SO_4 and concentration, the residue was purified by silica gel chromatography (Hexane:EtOAc = 1:1) to afford an orange powder with 93 % yield.

^{1}H NMR (500 MHz, CD_2Cl_2) δ 8.22 (d, *J* = 7.5 Hz, 1H), 7.84 (d, *J* = 8.7 Hz, 2H), 7.54 (d, *J* = 8.7 Hz, 3H), 7.44 (d, *J* = 7.6 Hz, 2H), 7.36 (d, *J* = 8.2 Hz, 1H), 7.34 (d, *J* = 4.7 Hz, 2H), 7.32 (d, *J* = 4.7 Hz, 2H), 7.26 (t, *J* = 7.6 Hz, 2H), 7.16 (d, *J* = 8.1 Hz, 1H), 6.82 (d, *J* = 9.0 Hz, 5H), 5.83 (s, 1H), 5.50 (d, *J* = 7.6 Hz, 1H), 4.31 (d, *J* = 4.1 Hz, 2H), 4.29 (d, *J* = 3.7 Hz, 1H), 3.74 (s, 3H), 3.74 (s, 3H), 3.65 (d, *J* = 9.5 Hz, 1H), 3.57 – 3.52 (m, 2H), 3.43 (d, *J* = 9.0 Hz, 1H), 2.71 (s, 3H), 2.41 (t, *J* = 6.4 Hz, 2H), 1.37 (s, 9H), 1.32 – 1.20 (m, 2H), 1.15 (d, *J* = 6.9 Hz, 6H), 1.13 (d, *J* = 6.7 Hz, 6H), 0.92 (s, 9H), 0.24 (s, 3H), 0.15 (s, 3H).

^{13}C NMR (126 MHz, CD_2Cl_2) δ 158.76, 158.73, 155.11, 154.84, 150.84, 150.80, 144.76, 141.97, 136.49, 135.39, 135.32, 131.87, 130.33, 130.28, 128.26, 127.83, 126.91, 126.05, 125.56, 122.69, 120.97, 117.49, 113.08, 90.88, 86.89, 81.28, 75.56, 70.15, 70.07, 61.42, 60.23, 58.31, 58.15, 55.17, 43.23, 43.13, 34.91, 30.97, 25.62, 24.67, 24.62, 24.47, 24.41, 20.18, 20.13, 17.97, 16.75, -4.65, -5.27.

^{31}P NMR (202 MHz, CD_2Cl_2) δ 149.04, 148.99.

HRMS(ESI$^+$) calculated for $C_{62}H_{80}N_7O_8PSi$ [MH^+]: 1110.5648 (calcd), 1110.5703 (found).

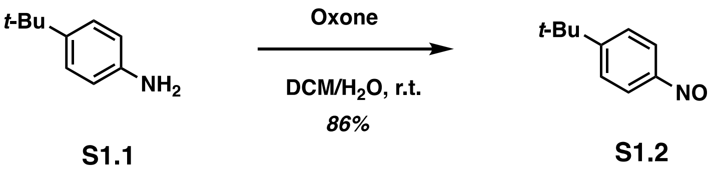

S1.1 S1.2

To a solution of the pure 4-*tert*-butylaniline (2 g, 13.4 mmol, 1.0 eq) in DCM was added the volume of an anaquas solution of Oxone (4 g, 2.0 eq per amine). The reaction was stirred at room temperature under Argon until the reaction completed. Extracted to separate the two phases and purified by silica gel chromatography (only Hexane) to give a green liquid with 86 % yield.

^{1}H NMR (500 MHz, $CDCl_3$) δ 7.85 (d, *J* = 8.5 Hz, 2H), 7.62 (d, *J* = 8.9 Hz, 2H), 1.38 (s, 9H).

^{13}C NMR (126 MHz, $CDCl_3$) δ 165.22, 159.79, 126.08, 121.00, 35.62, 30.95.
HRMS(ESI+) calculated for $C_{10}H_{13}NO$, [MH^+]: 164.1070 (calcd), 164.1086 (found).

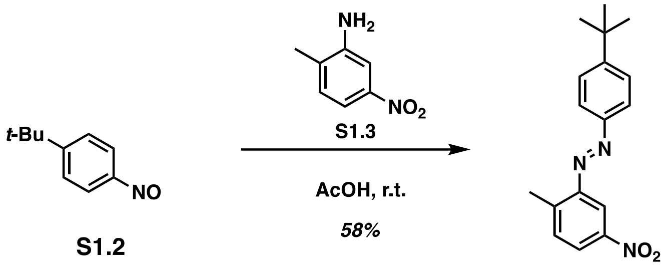

S1.2 S1.4

A mixture of 2-methyl-4-nitroaniline (2.5 g, 16 mmol, 1.0 eq) and 4-*tert*-butyl-1-nitrosobenzene (2.67 g, 16 mmol, 1.0 eq) dissolved in 20 mL acetic acid was stirred at room temperature for 2 days. The reaction was quenched by $NaHCO_3$ and followed by dilution with DCM and extraction with water. The organic layer was collected and dried over anhydrous Na_2SO_4. The solvent was removed by rotated evaporator and the crude product was purified by silica gel chromatography (Hexane:EtOAc = 20:1) to give 2.76 g product with 58 % yield.
^1H NMR (500 MHz, $CDCl_3$) δ 8.45 (d, *J* = 2.5 Hz, 1H), 8.15 (dd, *J* = 8.4, 2.5 Hz, 1H), 7.89 (d, *J* = 8.7 Hz, 2H), 7.56 (d, *J* = 8.7 Hz, 2H), 7.46 (d, *J* = 8.4 Hz, 1H), 2.78 (s, 3H), 1.40 (s, 9H).
^{13}C NMR (126 MHz, $CDCl_3$) δ 155.76, 150.63, 150.50, 147.06, 144.97, 131.91, 126.19, 124.09, 123.19, 110.79, 35.15, 31.25, 17.83.
HRMS(ESI+) calculated for $C_{17}H_{19}N_3O_2$ [MH^+]: 298.1550 (calcd), 298.1617 (found).

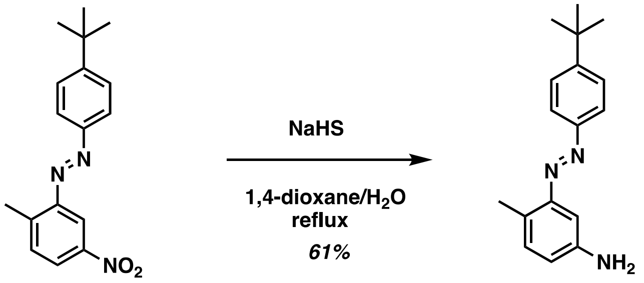

S1.4 2.4

The nitrobenzene (2.76 g, 9.3 mmol, 1.0 eq) was dissolved into a mixture of 140 mL 1,4-dioxane and 60 mL H_2O. Then, the reaction mixture was heated to 90 °C and the NaHS (1.4 g, 18.6 mmol, 2.0 eq) was added portion wise. The red suspension was allowed to stir for 2 hours at reflux, at which point the TLC monitoring indicated the reaction had completed. The reaction was quenched by saturated $NaHCO_3$ and the crude product was extracted by DCM (50 mL×3). The organic layers were combined and washed with H_2O. Then, the solvent was dried over anhydrous Na_2SO_4 and removed by rotary evaporator under reduced pressure. The orange crude product was purified by silica gel chromatography (only DCM) to give 1.52 g orange powder with 61 % yield.
^1H NMR (500 MHz, $CDCl_3$) δ 7.86 (d, *J* = 8.7 Hz, 2H), 7.53 (d, *J* = 8.7 Hz, 2H), 7.12 (d, *J* = 8.7 Hz, 1H), 7.01 (d, *J* = 2.6 Hz, 1H), 6.74 (dd, *J* = 8.1, 2.6 Hz, 1H), 3.83 (s, 2H), 2.61 (s, 3H), 1.38 (s, 9H).

^{13}C NMR (126 MHz, $CDCl_3$) δ 154.22, 151.23, 150.99, 144.57, 131.84, 128.81, 125.98, 122.66, 118.54, 101.60, 34.98, 31.30, 16.60.
HRMS(ESI$^+$) calculated for $C_{17}H_{21}N_3$ [MH$^+$]: 268.1808 (calcd), 268.1896 (found).

References

(1) Zheng, Y. Y.; Haruehanroengra, P.; Yadav, P. K.; Irani, S.; Mao, S.; Wang, T.; Hussain, M. M.; Sheng, j. Synthesis of Novel MicroRNA-30c Analogs to Reduce Apolipoprotein B Secretion in Human Hepatoma Cells. *Bio-Protoc.* **2022**, *12*, e4574.
(2) Abou-Elkhair, R. A. I.; Du, J.; Wasfy, A. A.; Khaill, N. A.; Maaroof, H. M.; Hassan, M. H.; Ahmed, A. S.; Hassan, A. E. A.; Sheng, J. Synthesis of 6-Aza-2-Hydroxyimino-5-Methylpyrimidine Nucleosides for Antiviral Evaluation. *Curr. Protoc.* **2021**, *1*, e329.
(3) Mathivanan, J.; Du, J.; Mao, S.; Zheng, Y. Y.; Sheng, J. Synthesis and Purification of N3-Methylcytidine (m3C) Modified RNA Oligonucleotides. *Curr. Protoc.* **2021**, *1*, e307.
(4) Chang, Z.; Mao, S.; Zheng, Y. Y.; Sheng, J. Synthesis and Functionality Study of Photoswitchable Hydrazone Oligodeoxynucleotides. *Curr. Protoc.* **2021**, *1*, e295.
(5) Mao, S.; Haruehanroengra, P.; Ranganathan, S. V.; Shen, F.; Begley, T. J.; Sheng, J. Base Pairing and Functional Insights into N3-Methylcytidine (m3C) in RNA. *ACS Chem. Biol.* **2021**, *16*, 76-85.
(6) Mao, S.; Sekula, B.; Ruszkowski, M.; Ranganathan, S. V.; Haruehanroengra, P.; Wu, Y.; Shen, F.; Sheng, J. Base pairing, structural and functional insights into N4-methylcytidine (m4C) and N4,N4-dimethylcytidine (m42C) modified RNA. *Nucleic Acids Res.* **2020**, *48*, 10087-10100.
(7) Wang, R.; Luo, Z.; He, K.; Delaney, M. O.; Chen, D.; Sheng, J. Base pairing and structural insights into the 5-formylcytosine in RNA duplex. *Nucleic Acids Res.* **2016**, *44*, 4968-4977.
(8) Case, D. A.; Cheatham III, T. E.; Darden, T.; Gohlke, H.; Luo, R.; Merz Jr., K. M.; Onufriev, A.; Simmerling, C.; Wang, B.; Woods, R. J. The Amber biomolecular simulation programs. *J. Comput. Chem.* **2005**, *26*, 1668-1688.
(9) Huang, H.; Chopra, R.; Verdine, G. L.; Harrison, S. C. Structure of a Covalently Trapped Catalytic Complex of HIV-1 Reverse Transcriptase: Implications for Drug Resistance. *Science* **1998**, *282*, 1669-1675.
(10) Case, D.; Betz, R.; Botello-Smith, W.; Cerutti, D.; Cheatham III, T.; Darden, T.; Duke, R.; Giese, T.; Gohlke, H.; Goetz, A. AmberTools 16. *University of California, San Francisco* **2016**.
(11) Hess, B.; Kutzner, C.; van der Spoel, D.; Lindahl, E. GROMACS 4: Algorithms for Highly Efficient, Load-Balanced, and Scalable Molecular Simulation. *J. Chem. Theory Comput.* **2008**, *4*, 435-447.
(12) He, W.; Naleem, N.; Kleiman, D.; Kirmizialtin, S. Refining the RNA Force Field with Small-Angle X-ray Scattering of Helix–Junction–Helix RNA. *J. Phys. Chem. Lett.* **2022**, *13*, 3400-3408.
(13) Maier, J. A.; Martinez, C.; Kasavajhala, K.; Wickstrom, L.; Hauser, K. E.; Simmerling, C. ff14SB: Improving the Accuracy of Protein Side Chain and Backbone Parameters from ff99SB. *J. Chem. Theory Comput.* **2015**, *11*, 3696-3713.
(14) Zgarbová, M.; Otyepka, M.; Šponer, J.; Mládek, A.; Banáš, P.; Cheatham, T. E., III; Jurečka, P. Refinement of the Cornell et al. Nucleic Acids Force Field Based on Reference Quantum Chemical Calculations of Glycosidic Torsion Profiles. *J. Chem. Theory Comput.* **2011**, *7*, 2886-2902.

(15) Jorgensen, W. L.; Chandrasekhar, J.; Madura, J. D.; Impey, R. W.; Klein, M. L. Comparison of simple potential functions for simulating liquid water. *J. Chem. Phys.* **1983**, *79*, 926-935.
(16) Yoo, J.; Aksimentiev, A. Improved Parametrization of Li+, Na+, K+, and Mg2+ Ions for All-Atom Molecular Dynamics Simulations of Nucleic Acid Systems. *J. Phys. Chem. Lett.* **2012**, *3*, 45-50.
(17) Wang, J.; Wolf, R. M.; Caldwell, J. W.; Kollman, P. A.; Case, D. A. Development and testing of a general amber force field. *J. Comput. Chem.* **2004**, *25*, 1157-1174.
(18) Dupradeau, F.-Y.; Pigache, A.; Zaffran, T.; Savineau, C.; Lelong, R.; Grivel, N.; Lelong, D.; Rosanski, W.; Cieplak, P. The R.E.D. tools: advances in RESP and ESP charge derivation and force field library building. *Phys. Chem. Chem. Phys.* **2010**, *12*, 7821-7839.
(19) Meagher, K. L.; Redman, L. T.; Carlson, H. A. Development of polyphosphate parameters for use with the AMBER force field. *J. Comput. Chem.* **2003**, *24*, 1016-1025.
(20) Vanquelef, E.; Simon, S.; Marquant, G.; Garcia, E.; Klimerak, G.; Delepine, J. C.; Cieplak, P.; Dupradeau, F.-Y. R.E.D. Server: a web service for deriving RESP and ESP charges and building force field libraries for new molecules and molecular fragments. *Nucleic Acids Res.* **2011**, *39*, W511-W517.
(21) Kuznetsova, A. A.; Tyugashev, T. E.; Alekseeva, I. V.; Timofeyeva, N. A.; Fedorova, O. S.; Kuznetsov, N. A. Insight into the mechanism of DNA synthesis by human terminal deoxynucleotidyltransferase. *Life Sci. Alliance* **2022**, *5*, e202201428.
(22) Allnér, O.; Nilsson, L.; Villa, A. Magnesium Ion–Water Coordination and Exchange in Biomolecular Simulations. *J. Chem. Theory Comput.* **2012**, *8*, 1493-1502.
(23) Hockney, R. W.; Goel, S. P.; Eastwood, J. W. Quiet high-resolution computer models of a plasma. *J. Comput. Phys.* **1974**, *14*, 148-158.
(24) Hess, B.; Bekker, H.; Berendsen, H. J. C.; Fraaije, J. G. E. M. LINCS: A linear constraint solver for molecular simulations. *J. Comput. Chem.* **1997**, *18*, 1463-1472.
(25) Miyamoto, S.; Kollman, P. A. Settle: An analytical version of the SHAKE and RATTLE algorithm for rigid water models. *J. Comput. Chem.* **1992**, *13*, 952-962.
(26) Darden, T.; York, D.; Pedersen, L. Particle mesh Ewald: An N·log(N) method for Ewald sums in large systems. *J. Chem. Phys.* **1993**, *98*, 10089-10092.
(27) Bussi, G.; Donadio, D.; Parrinello, M. Canonical sampling through velocity rescaling. *J. Chem. Phys.* **2007**, *126*.
(28) Parrinello, M.; Rahman, A. Polymorphic transitions in single crystals: A new molecular dynamics method. *J. Appl. Phys.* **1981**, *52*, 7182-7190.
(29) Dangerfield, T. L.; Kirmizialtin, S.; Johnson, K. A. Conformational dynamics during misincorporation and mismatch extension defined using a DNA polymerase with a fluorescent artificial amino acid. *J. Biol. Chem.* **2022**, *298*.
(30) Me, F.; Trucks, G.; Schlegel, H.; Scuseria, G.; Robb, M.; Cheeseman, J.; Scalmani, G.; Barone, V.; Petersson, G.; Nakatsuji, H. Gaussian 16, revision c. 01. Gaussian. *Inc., Wallingford CT* **2016**.
(31) Humphrey, W.; Dalke, A.; Schulten, K. VMD: Visual molecular dynamics. *J. Mol. Graph.* **1996**, *14*, 33-38.

Orbitrap MS Analysis Spectrum

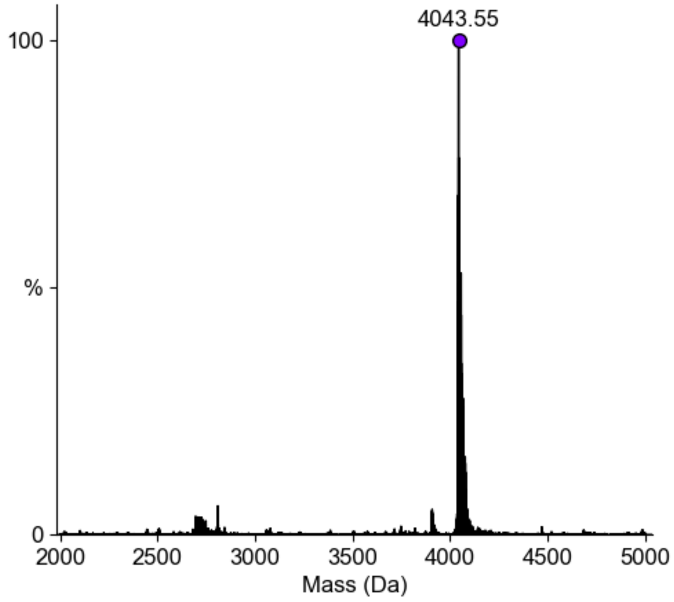

Orbitrap MS of #1, 12mer in Scheme 3 (5′ -AAUGCC*GCACUG-3′) [M-H]=4043.55 (calc. 4043.51).

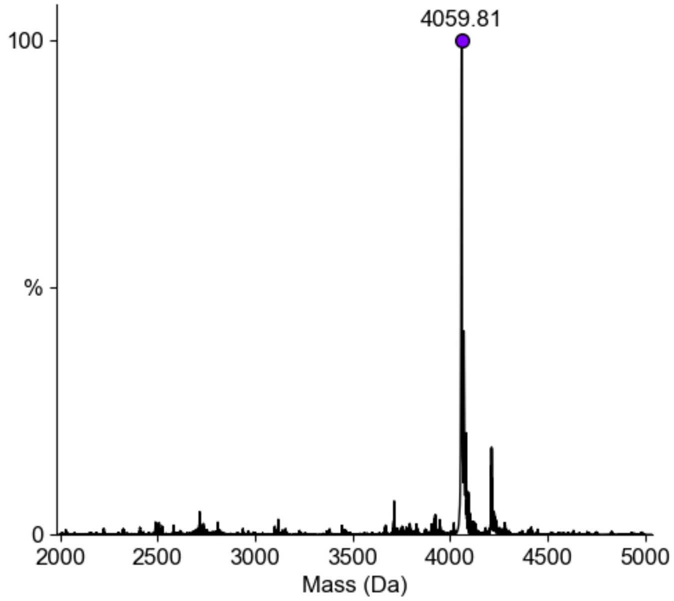

Orbitrap MS of #2, 12mer in Scheme 3 (5′ -GGACUC*CUGCAG-3′) [M-H]=4059.81 (calc. 4059.51).

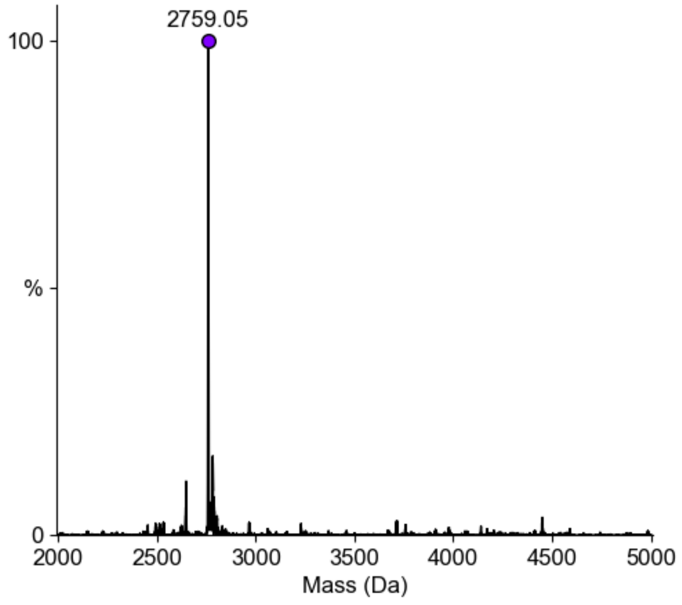

Orbitrap MS of #3, 8mer in Scheme 3 (5′ -UC*GUACGA-3′) [M-H]=2759.05 (calc. 2758.73).

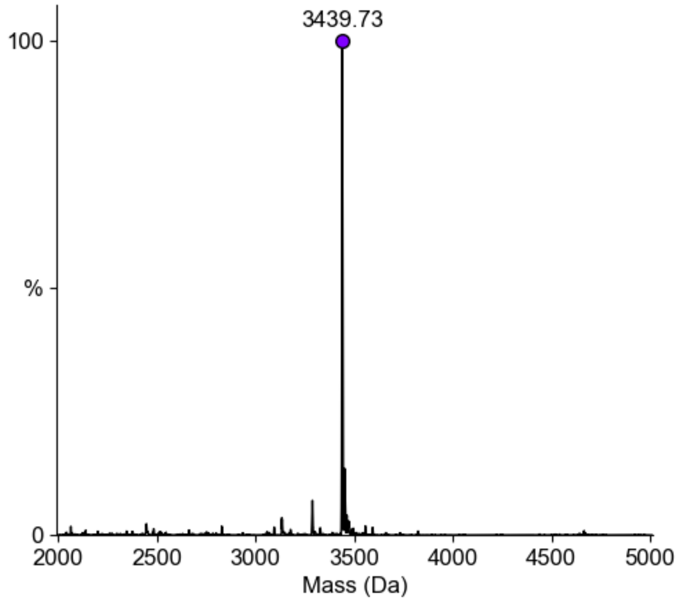

Orbitrap MS of #4, 10mer in Scheme 3 (5′ -CCGGC*GCCGG-3′) [M-H]=3439.73 (calc. 3439.15).

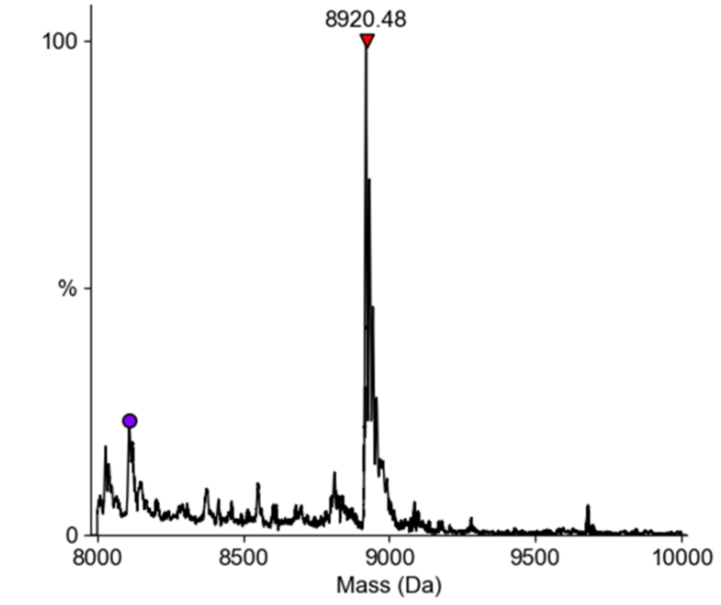

Orbitrap MS of #5, 27mer in Scheme 3 (5′ -GAAC*GCUAUGAGGACAUGGCAGCCUUC-3′) [M-H]=8920.48 (calc. 8920.45).

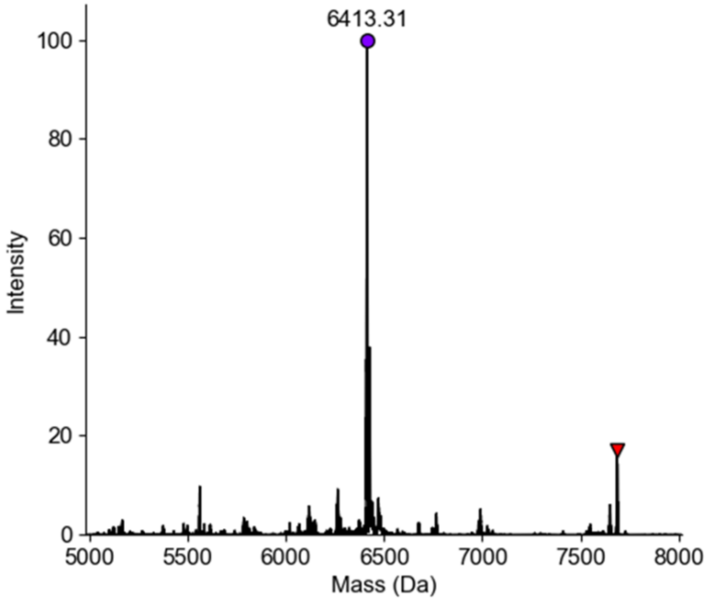

Orbitrap MS of #6 19mer in Scheme 3 (5′ -GAAC*GCUAUGAGGACAUGG-3′) [M-H]=6413.31 (calc. 6413.96).

Copies of Related NMR Spectra

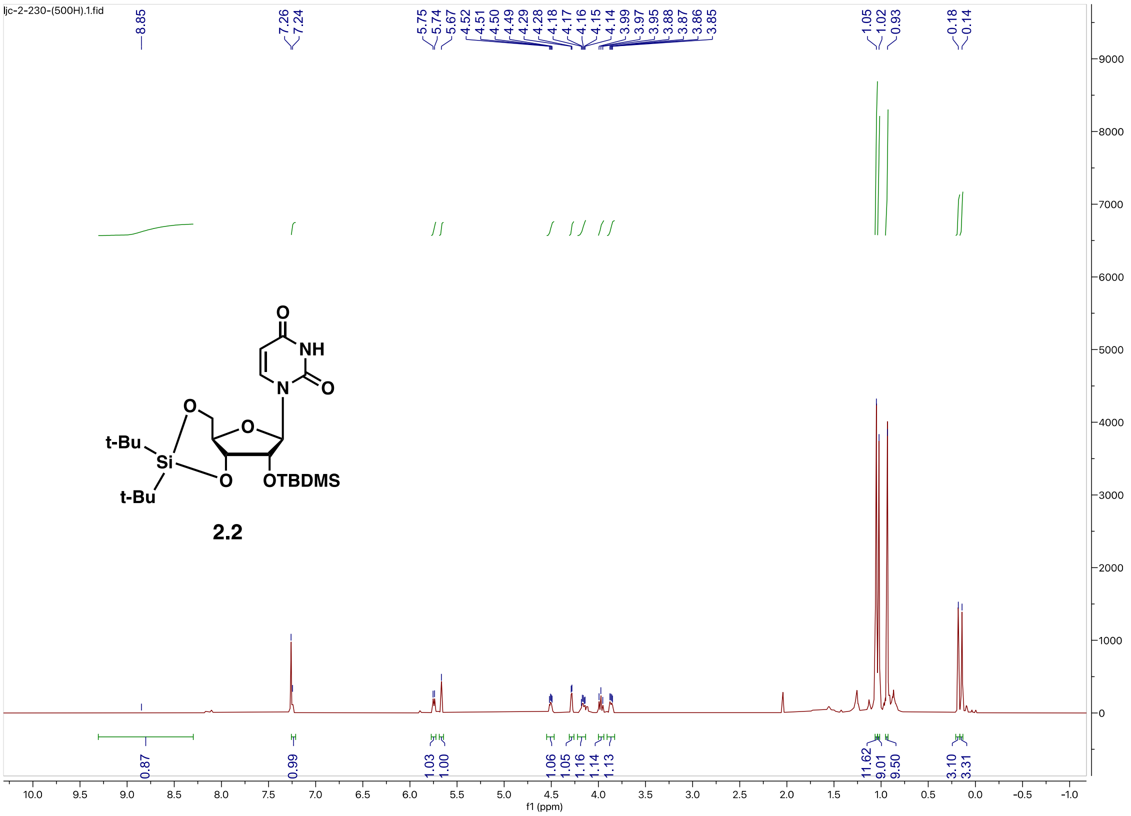

500 MHz, ^{1}H-NMR of Compound 2.2 in $CDCl_3$

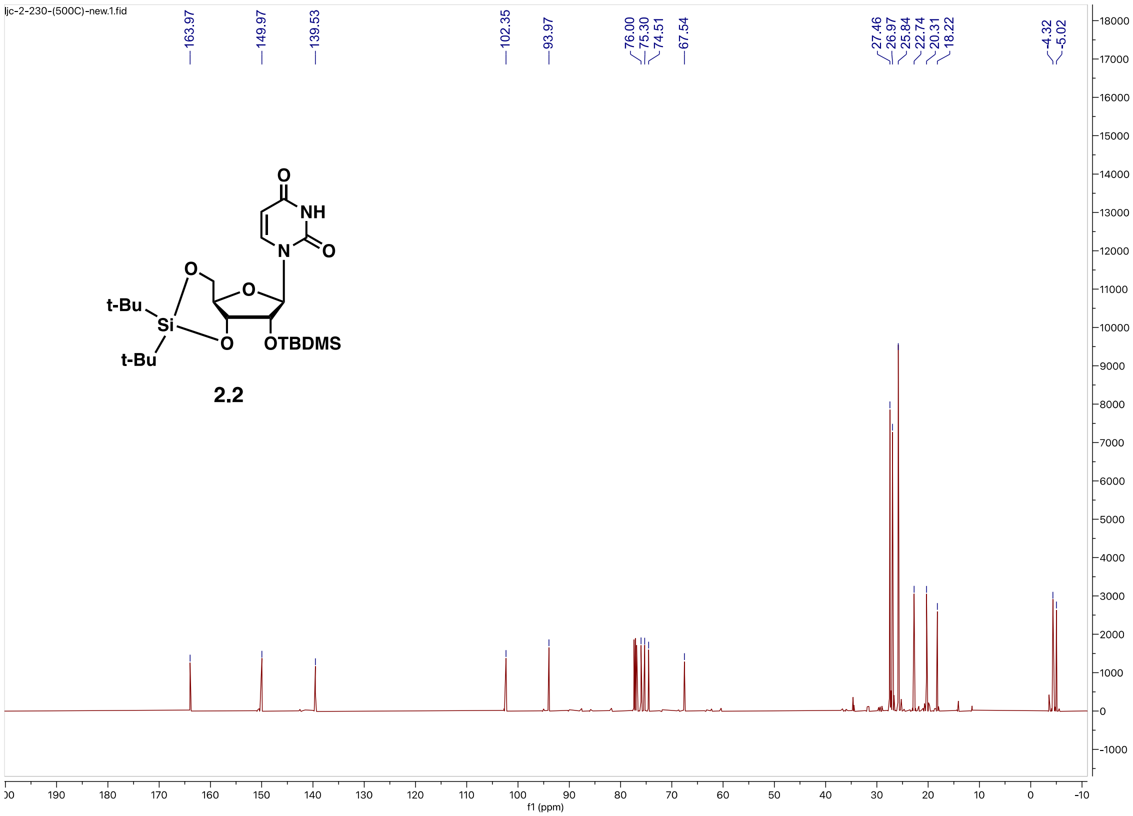

126 MHz, ^{13}C-NMR of Compound 2.2 in $CDCl_3$

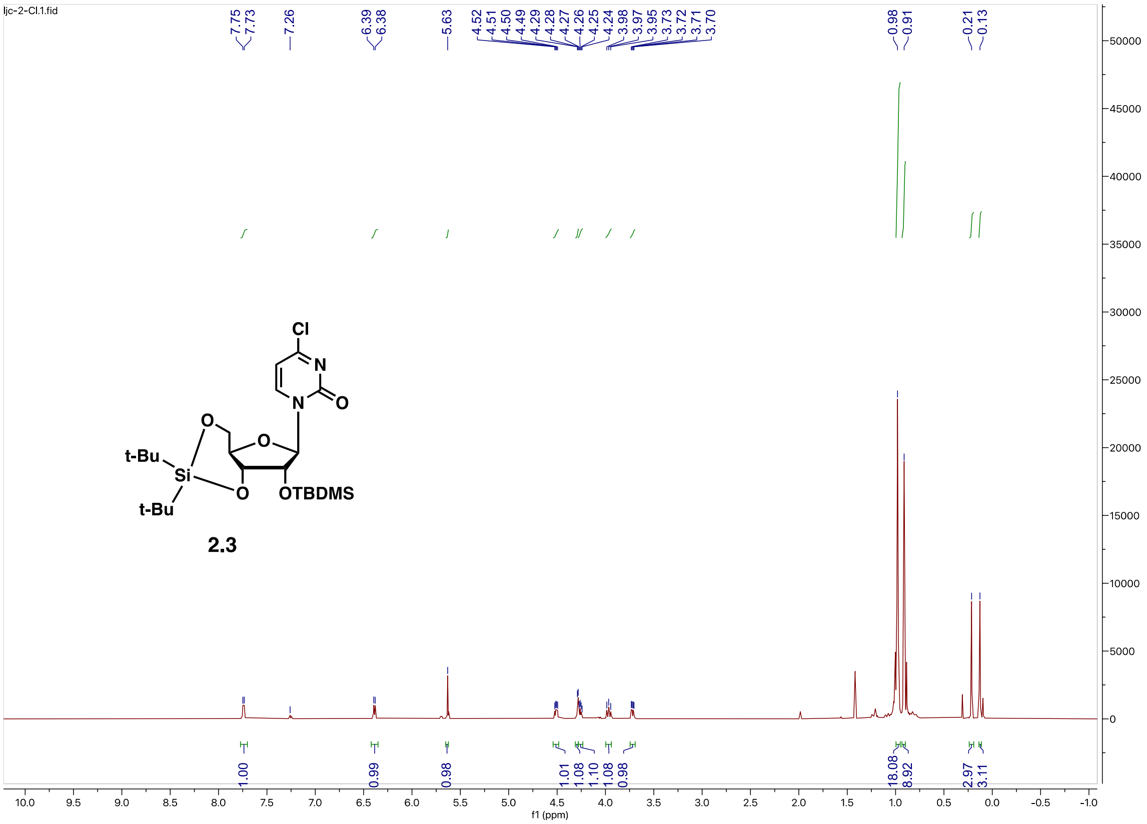

500 MHz, ^{1}H-NMR of Compound 2.3 in $CDCl_3$

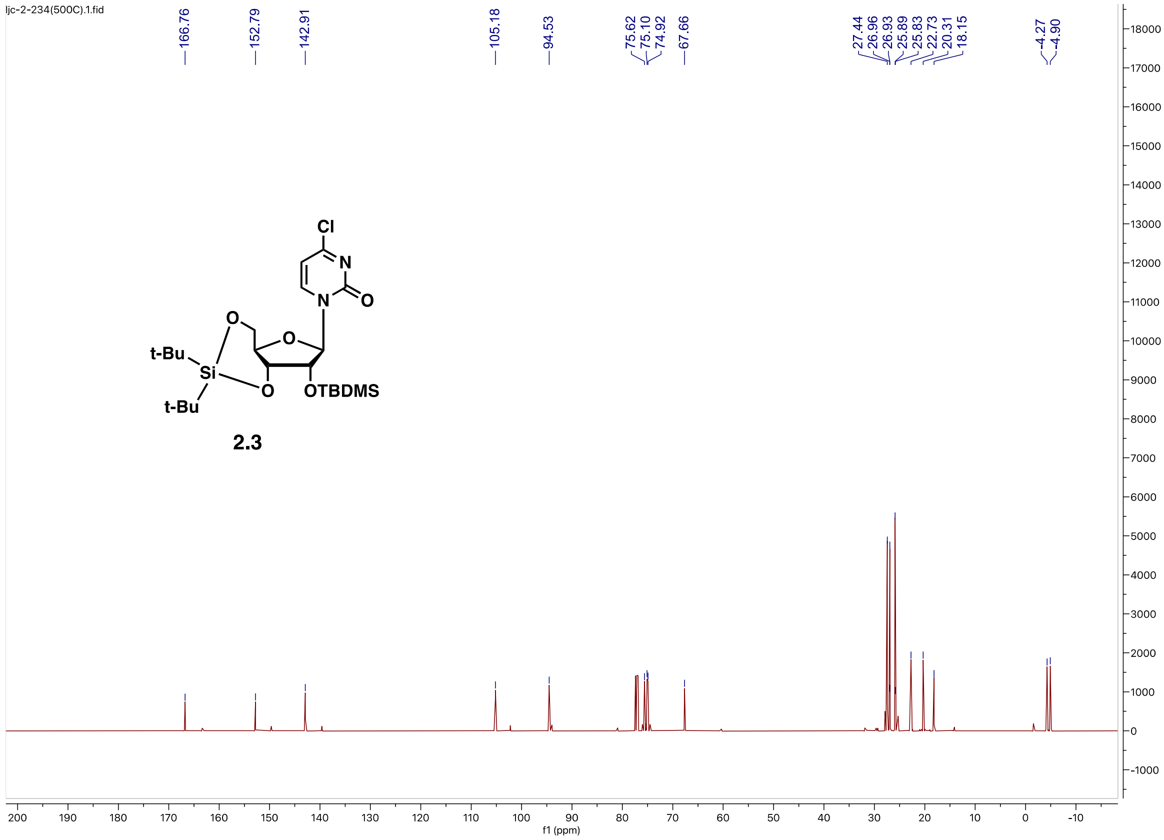

126 MHz, ^{13}C-NMR of Compound 2.3 in $CDCl_3$

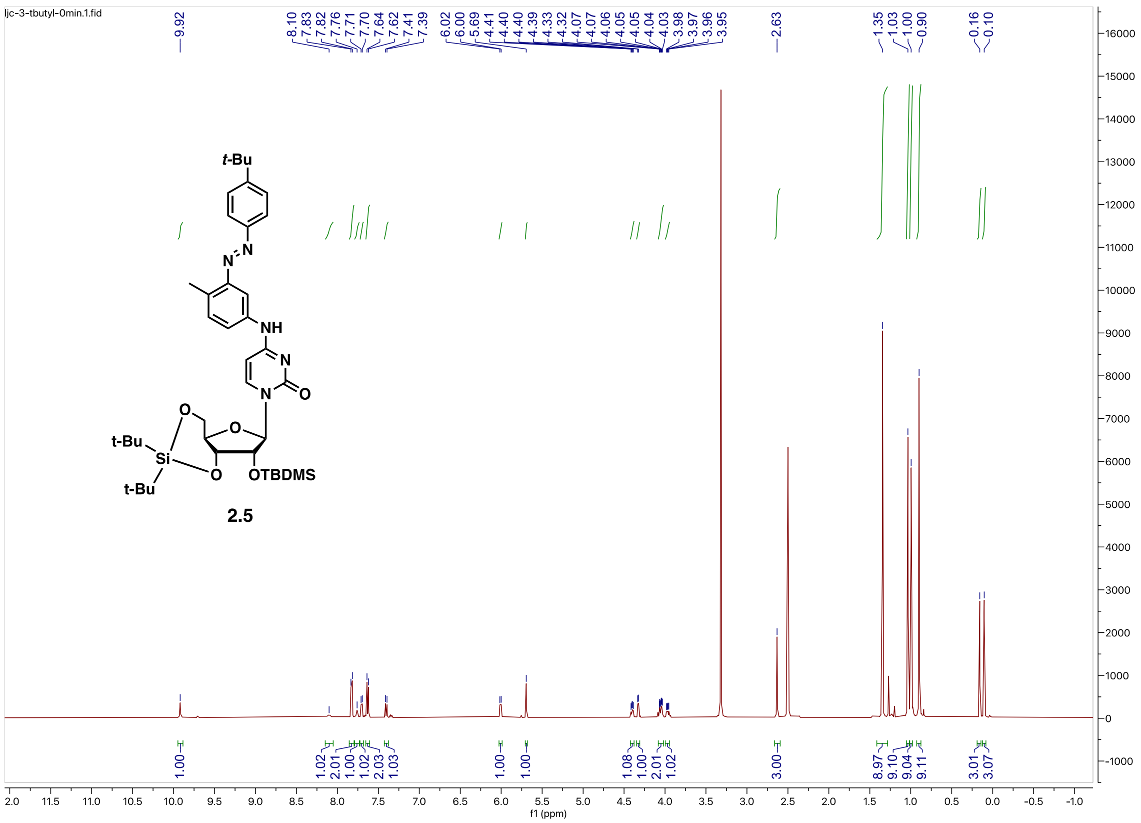

500 MHz, ^{1}H-NMR of Compound 2.5 in DMSO-d_6

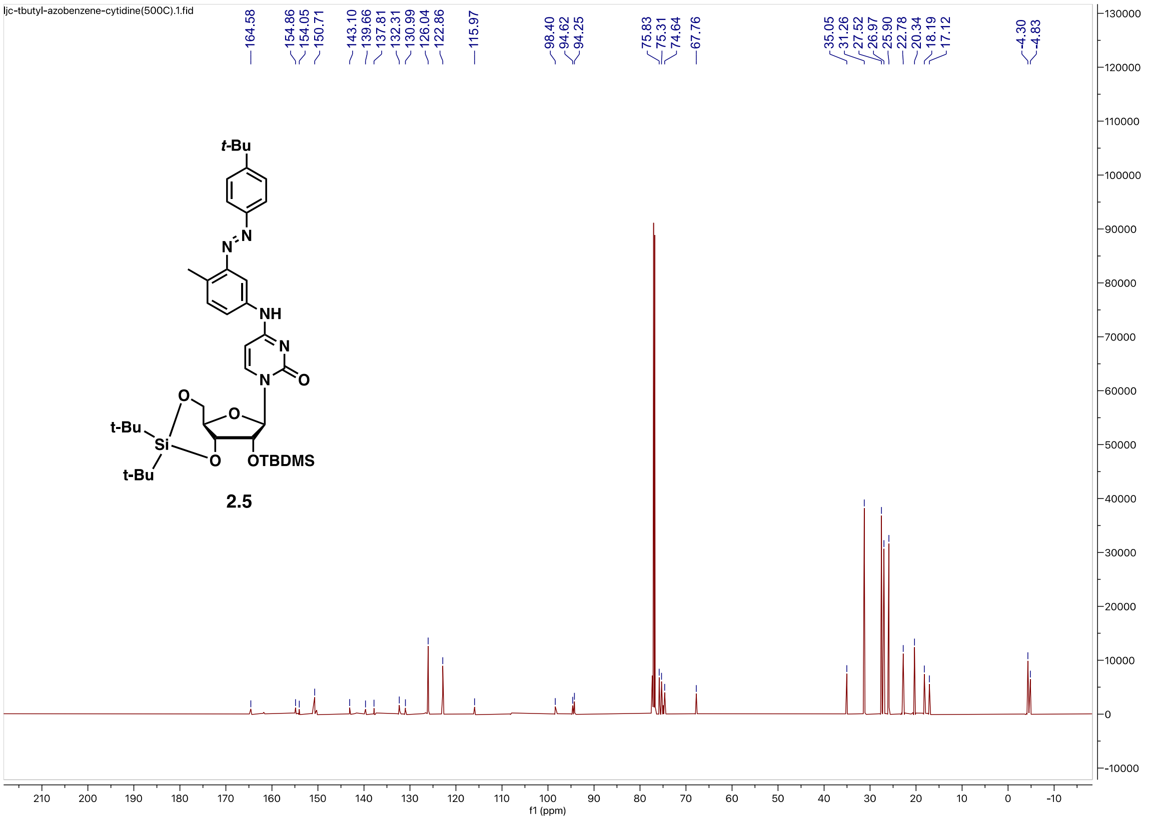

126 MHz, ^{13}C-NMR of Compound 2.5 in DMSO-d_6

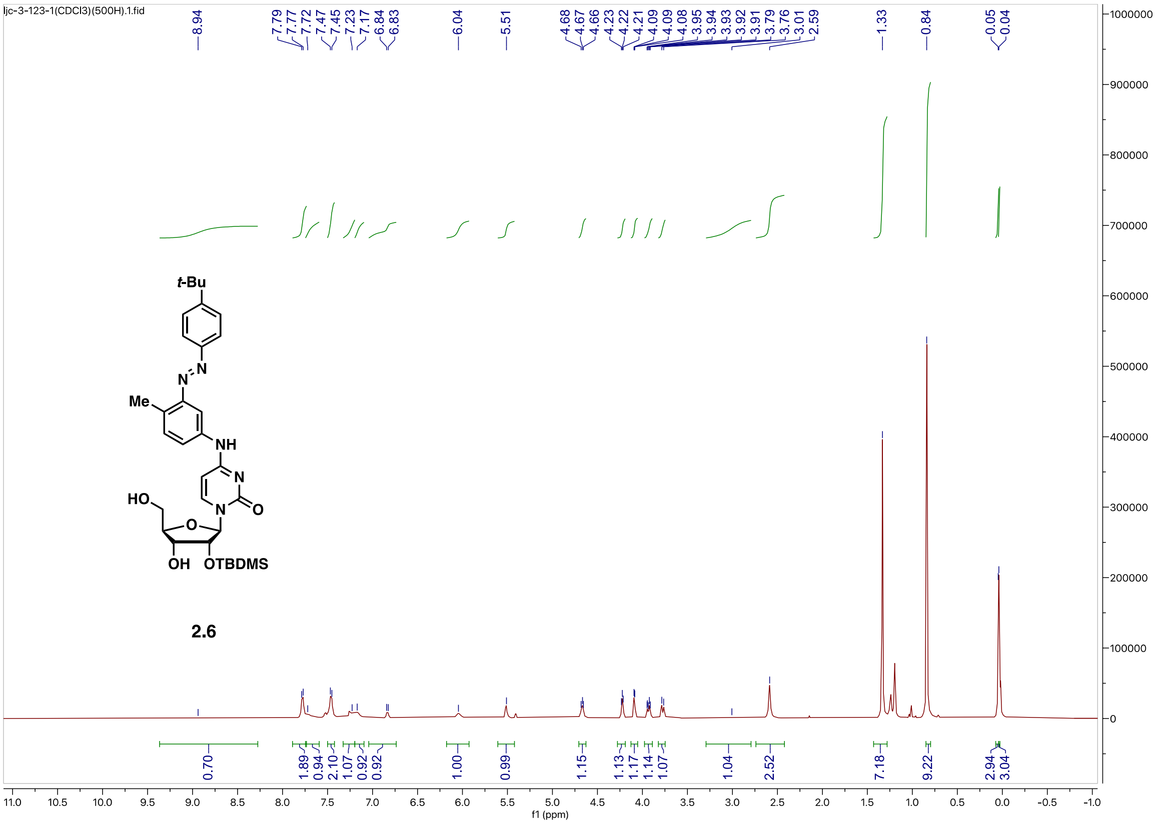

2.6

500 MHz, ¹H-NMR of Compound 2.6 in $CDCl_3$

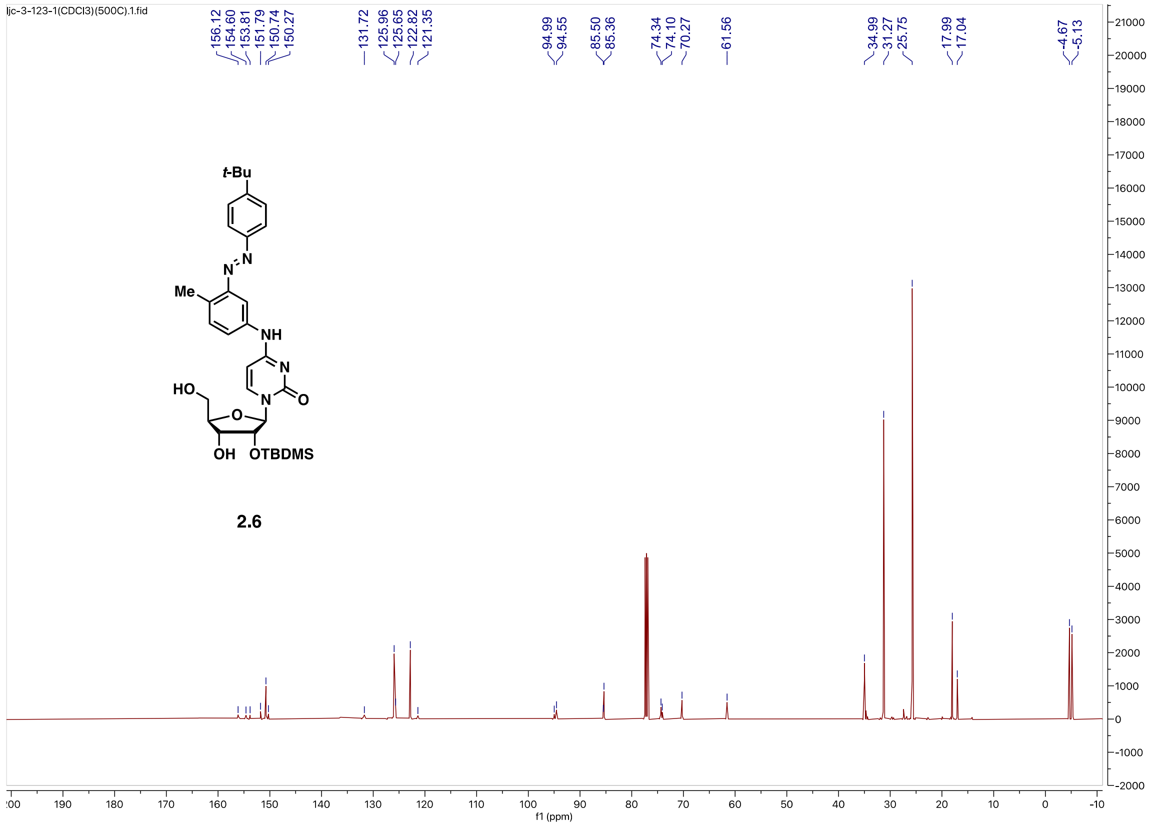

126 MHz, ^{13}C-NMR of Compound 2.6 in $CDCl_3$

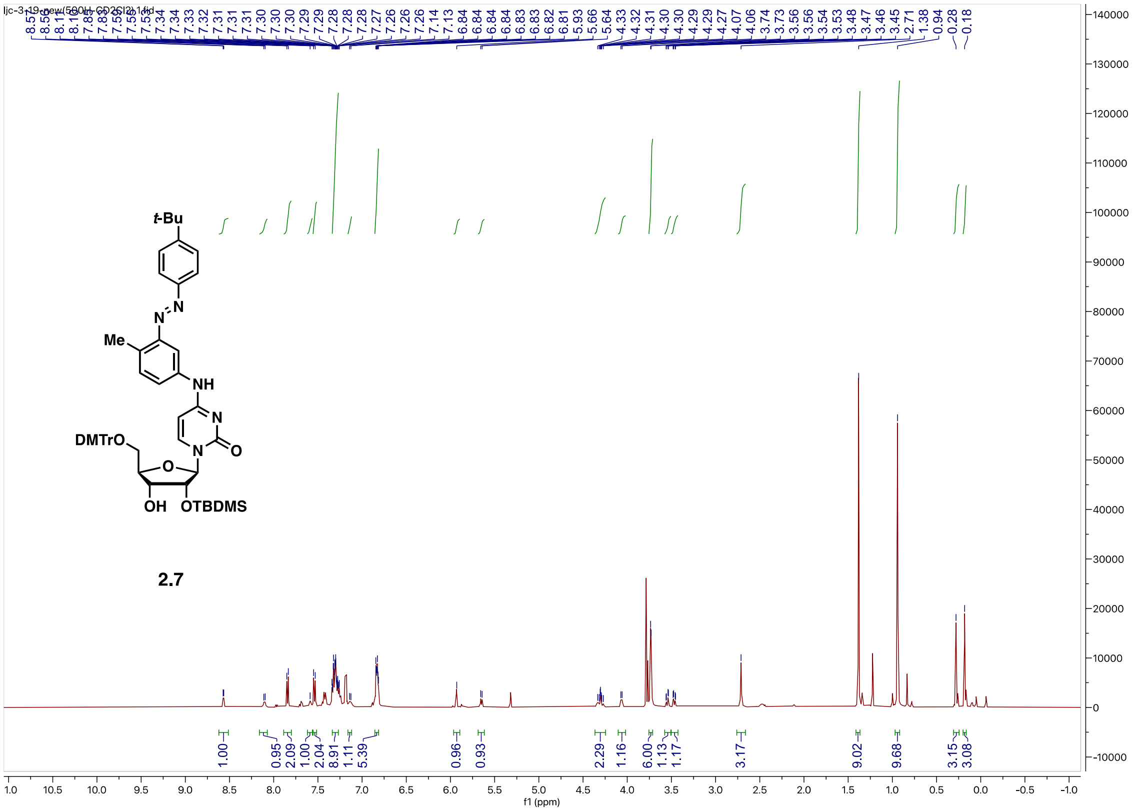

500 MHz, ^{1}H-NMR of Compound 2.7 in CD_2Cl_2

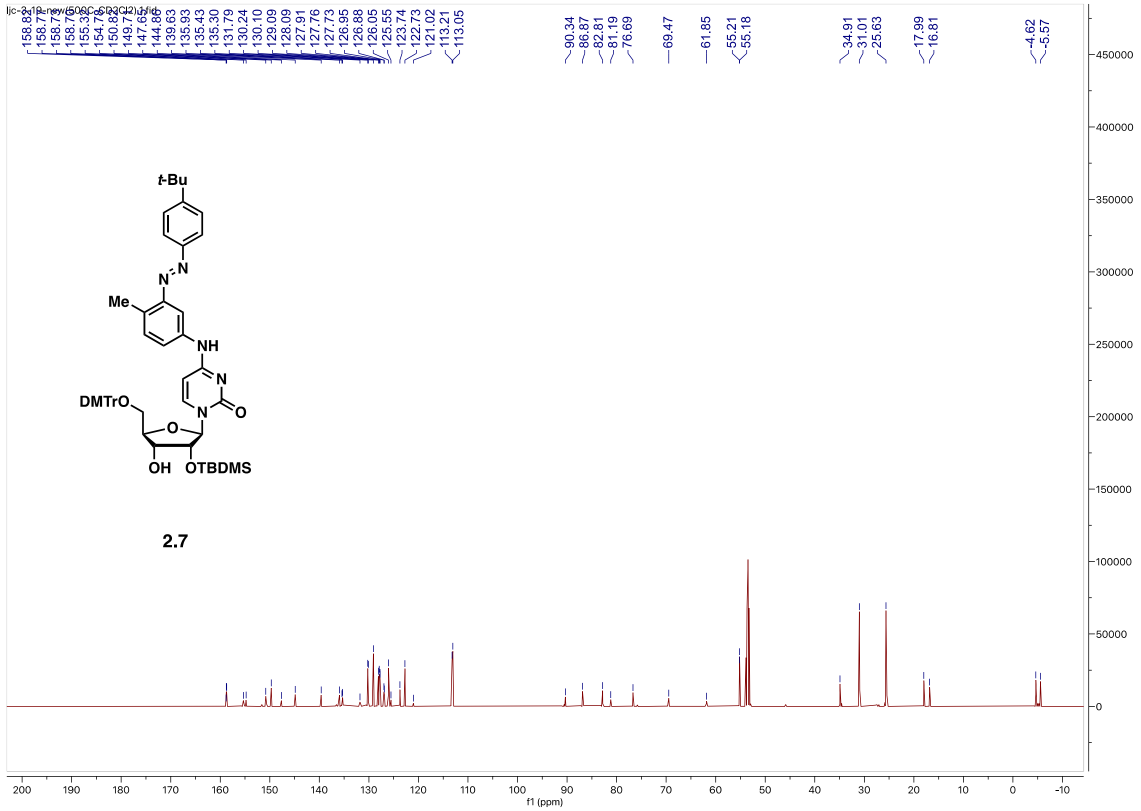

126 MHz, ^{13}C-NMR of Compound 2.7 in CD_2Cl_2

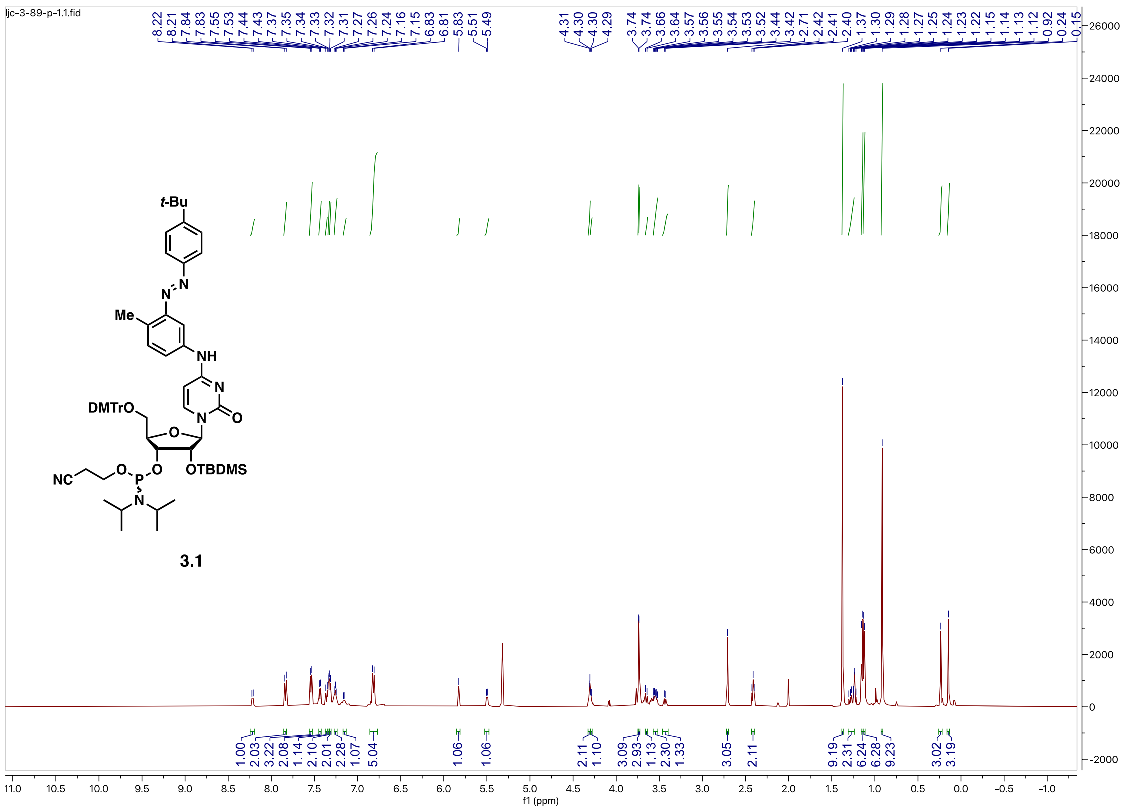

500 MHz, ^1H-NMR of Compound 3.1 in CD_2Cl_2

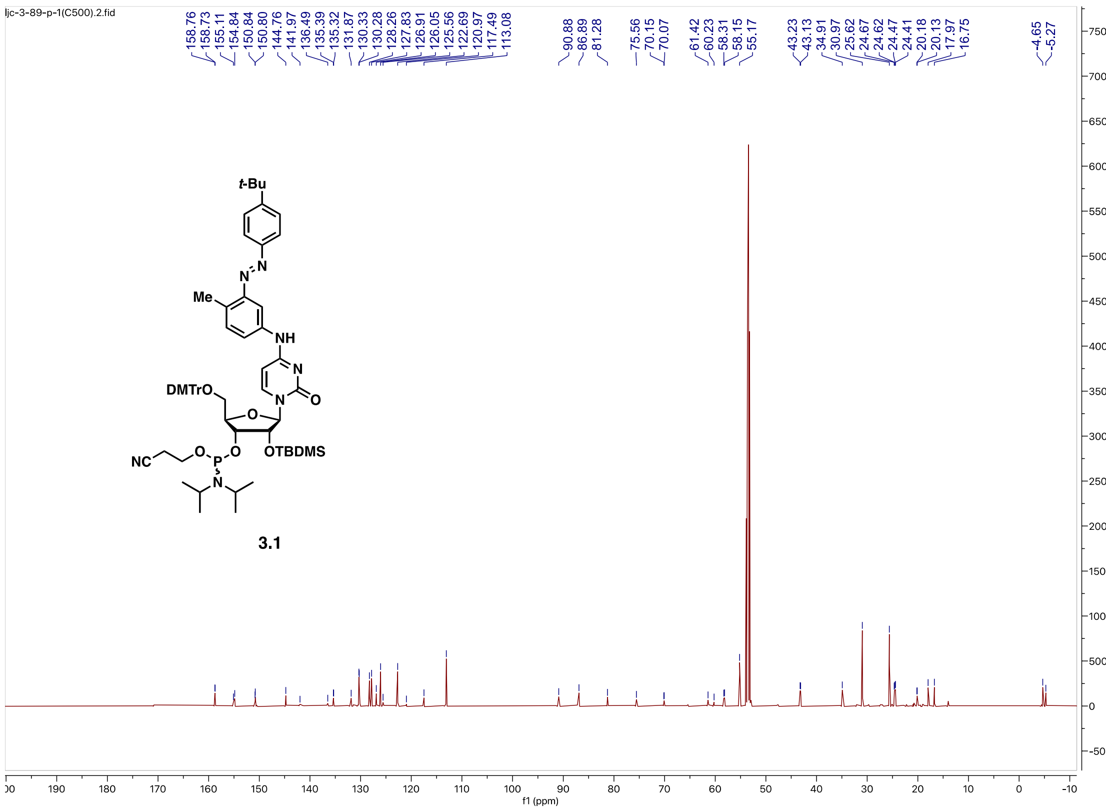

126 MHz, ^{13}C-NMR of Compound 3.1 in CD_2Cl_2

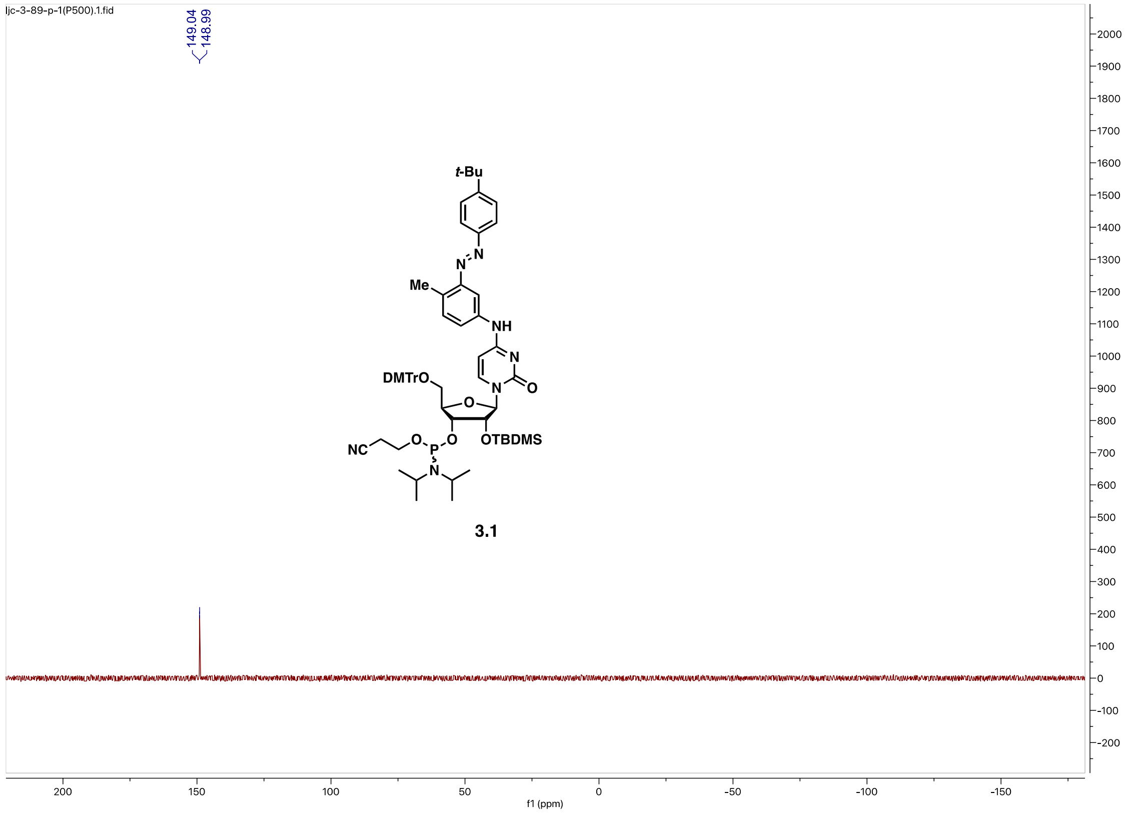

202 MHz, ^{31}P-NMR of Compound 3.1 in CD_2Cl_2

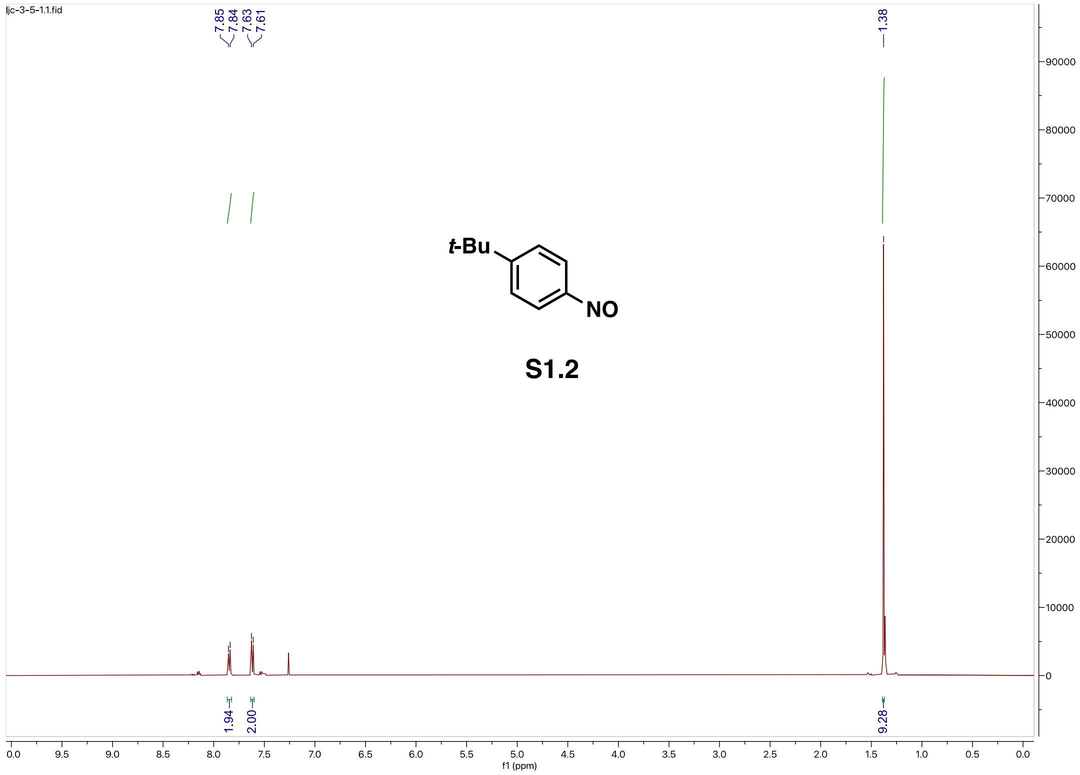

500 MHz, ^{1}H-NMR of Compound S1.2 in $CDCl_3$

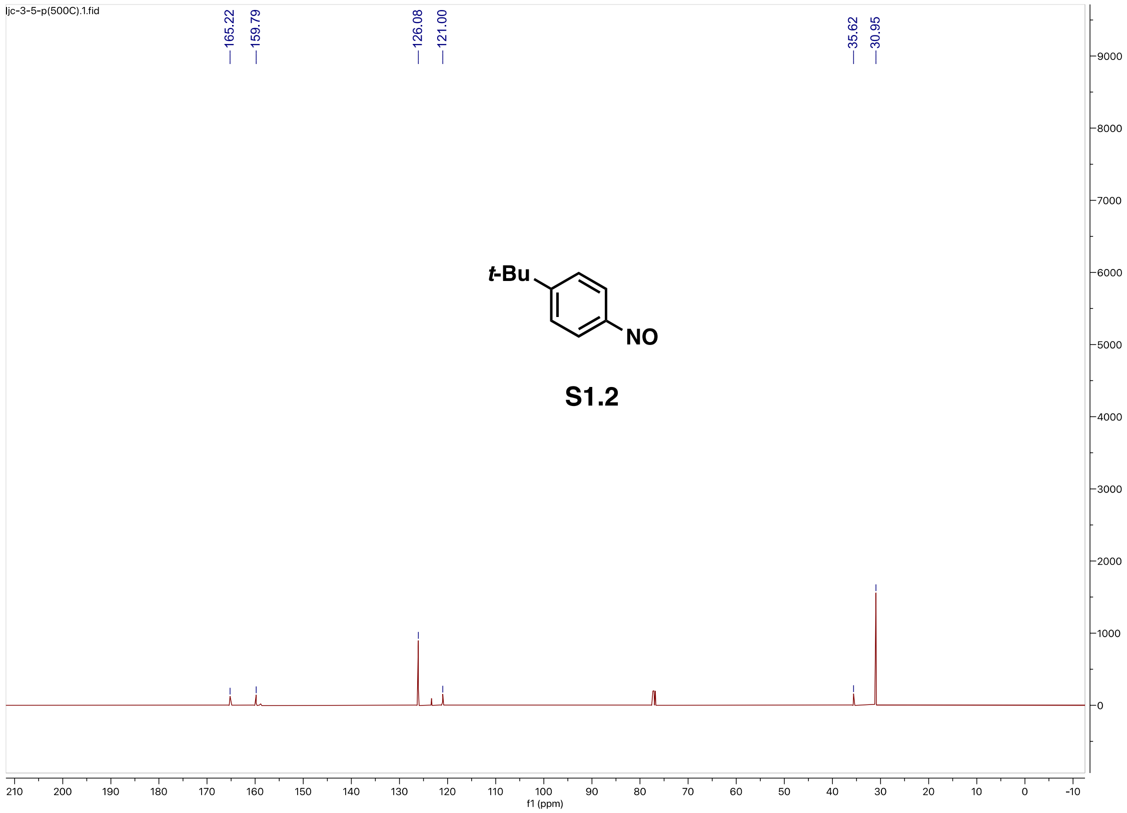

126 MHz, ^{13}C-NMR of Compound S1.2 in $CDCl_3$

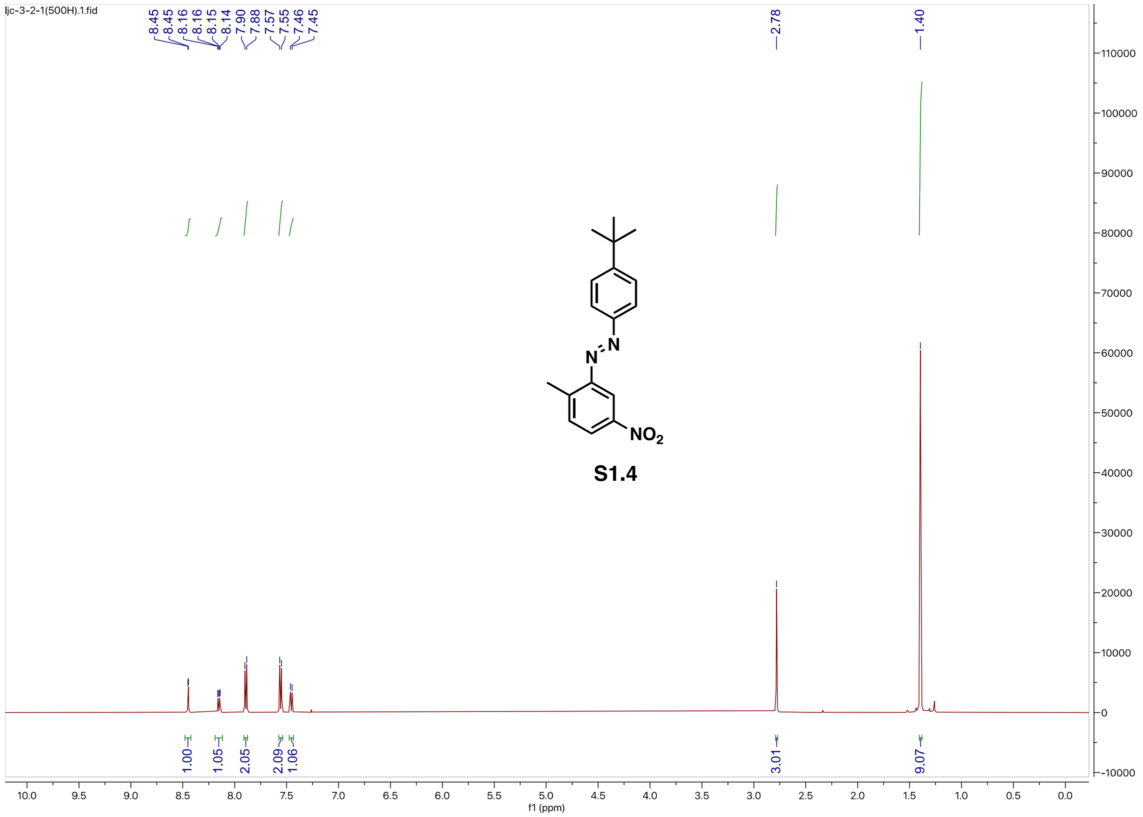

500 MHz, ^{1}H-NMR of Compound S1.4 in $CDCl_3$

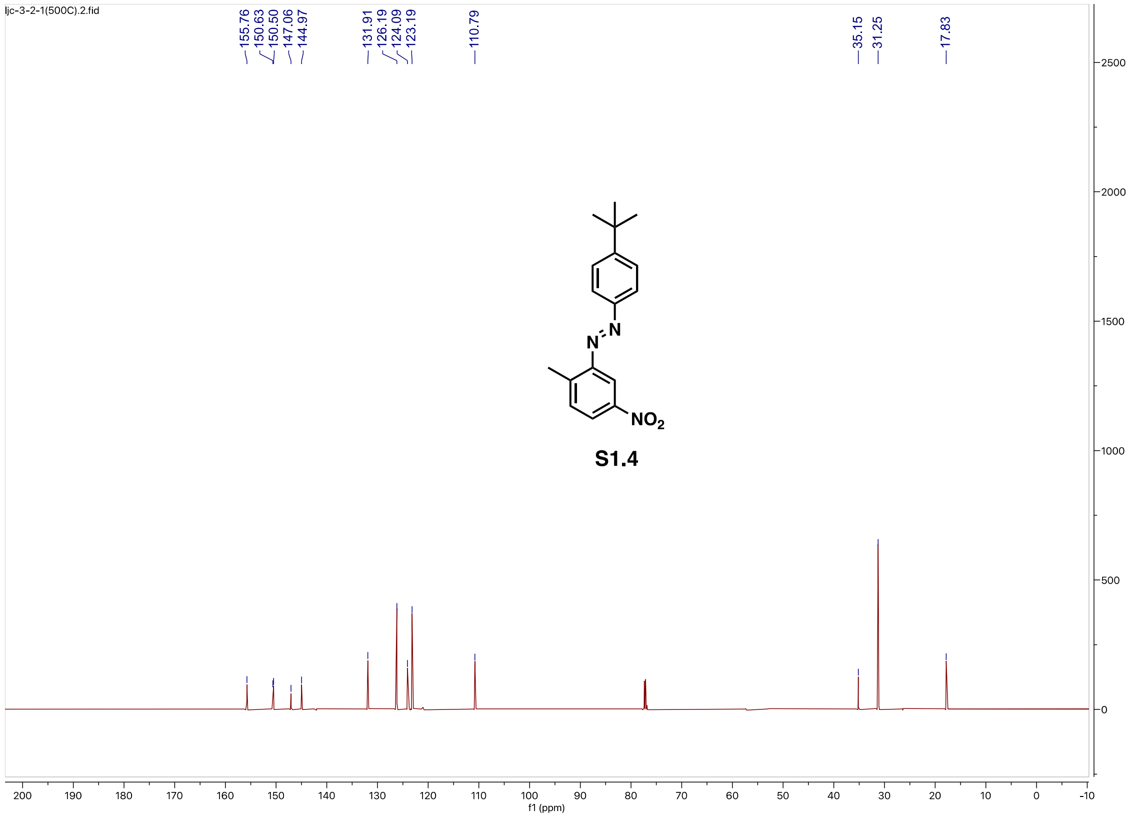

126 MHz, ^{13}C-NMR of Compound S1.4 in $CDCl_3$

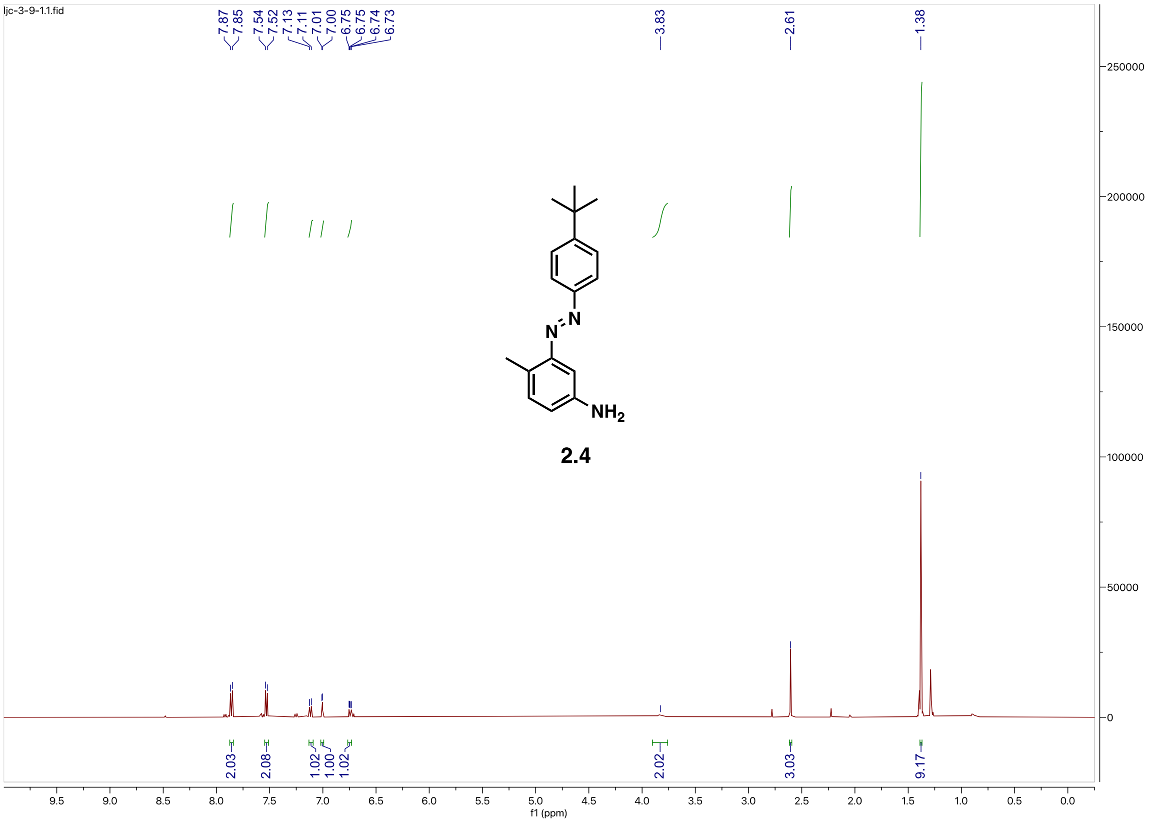

500 MHz, ^{1}H-NMR of Compound 2.4 in $CDCl_3$

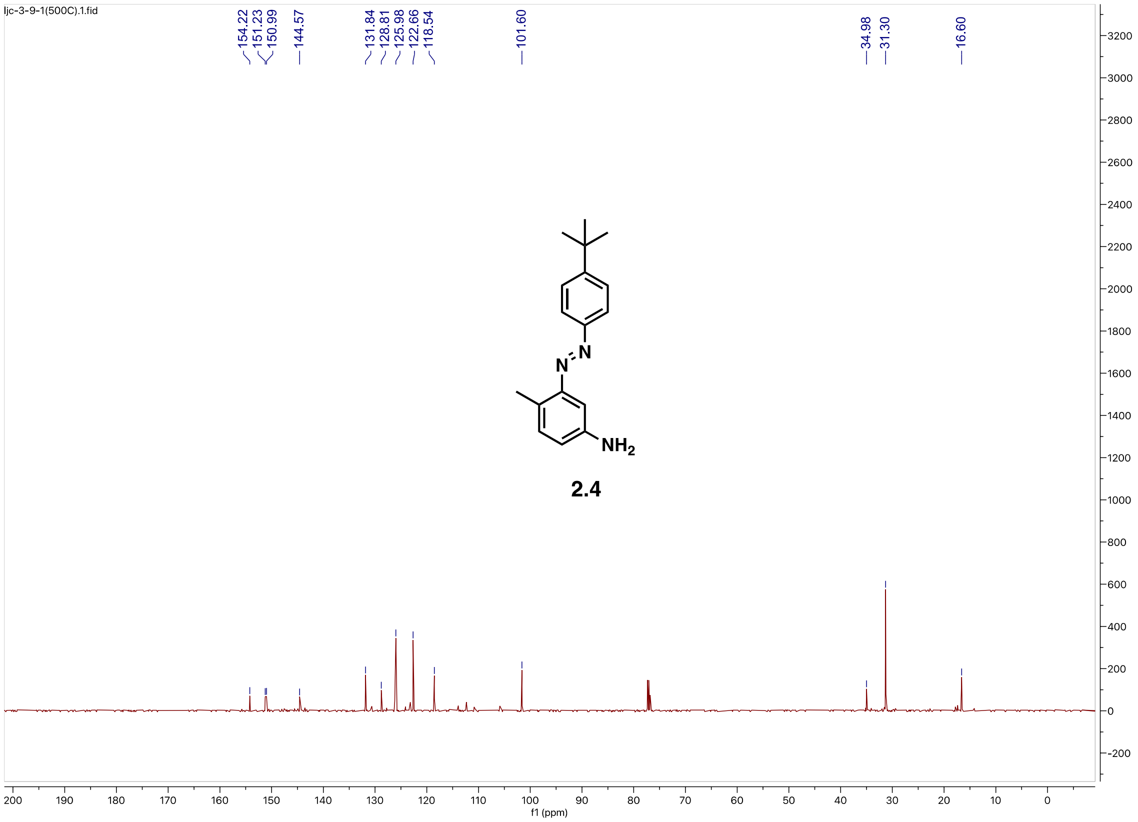

126 MHz, ^{13}C-NMR of Compound 2.4 in $CDCl_3$
